# Sustainable tea plantations: Harnessing chemical-microbial synergy and smart application triangulation for targeted weed control

**DOI:** 10.1016/j.jare.2025.10.054

**Published:** 2025-10-30

**Authors:** Lan Chen, Xiaolong Yang, Zhongzeng Su, Xiong Guan, Zixuan Wang, Tianpei Huang

**Affiliations:** State Key Laboratory of Agricultural and Forestry Biosecurity & Key Laboratory of Biopesticide and Chemical Biology of Ministry of Education & Biopesticide Research Center, College of Life Sciences, Fujian Agriculture and Forestry University, Fuzhou 350002, China

**Keywords:** Tea plantation weeds, Multidimensional hazards, Chemical herbicides, Microbial herbicides, Herbicide performance, Sustainable weed management

## Abstract

**Background:**

Over 200 weed species—predominantly from the Poaceae and Asteraceae families—infest tea plantations. These weeds compete aggressively with tea plants for essential resources (light, water, and nutrients), serve as reservoirs for pathogens and pests, and bioaccumulate phytotoxic compounds (e.g., pyrrolizidine alkaloids and heavy metals), jeopardizing agroecosystem health. Although chemical herbicides are currently the most effective and economical method, their long-term overuse poses ecological risks and threatens agricultural sustainability, highlighting an urgent need for sustainable alternatives. Although chemical herbicides remain the most effective and economical method, their long-term overuse causes ecological risks and challenges agricultural sustainability.

**Aim of review:**

This review synthesizes current knowledge on sustainable weed management in tea plantations by evaluating the strengths and limitations of chemical and microbial herbicides. It further advances the novel concepts of chemical–microbial synergy and smart application triangulation—integrated strategies not explicitly addressed in previous reviews—that provide new directions for developing next-generation sustainable weed control.

**Key scientific concepts of review:**

This review systematically examines three critical aspects of weed management in tea plantations: (1) characterizing weed biodiversity and assessing multidimensional threats through taxonomic profiling of invasive species, with particular focus on their cascading impacts on tea agroecosystems including resource competition, disease transmission, and soil toxicity; (2) evaluating the mechanisms and trade-offs of current herbicide approaches through comparative analysis of chemical herbicides (noting their efficiency but ecological concerns) and microbial herbicides (considering their environmental benefits but field performance variability); (3) exploring sustainable optimization strategies by investigating innovative approaches such as precision herbicide formulations (nano-formulations, smart delivery systems) and integrated weed management solutions (microbiome engineering, epigenome editing).

## Highlights

• The taxonomic diversity and multidimensional hazards of weeds in tea plantations.
• The current state of development and application of herbicides.
• Mechanistic insights into the efficacy of herbicides.
• Current herbicide development and application strategies.
• Smart sustainable strategies to enhance herbicide efficiency and weed control efficacy.

## Graphical abstract

**Figure:**
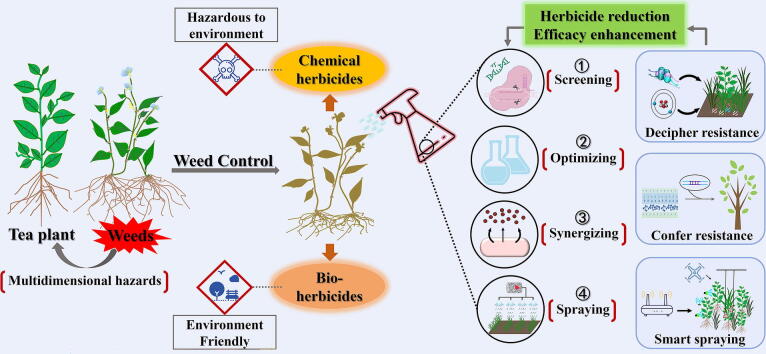

## Introduction

Tea plant (*Camellia sinensis*), an evergreen shrub of Theaceae family, is an important globally cultivated cash crop. Farmers harvest tender tea leaves in spring and autumn, and these leaves are then processed into tea products rich in bioactive compounds such as tea polyphenols, theanine, and caffeine. These components not only determine tea quality but also confer significant health benefits [1].

Currently, tea production faces substantial challenges from biotic stressors. Insect pests and diseases significantly reduce the yield and quality of tea. Among them, major fungal diseases include anthracnose, leaf spot, and ring spot, while key insect pests include tea geometrid, tea green leafhopper, and green leaf bug [2,3]. It should be noted that weeds, which are the most prevalent non-crop plants in tea plantations, also exert multifaceted ecological impacts. They compete with tea plants for water, fertilizers, and nutritional resources, while serving as reservoirs for pests and pathogens, thereby indirectly exacerbating biotic stresses in the tea plantation [4]. Significantly, recent studies highlight weeds as potential vectors of heavy metal contamination, such as lead (Pb), cadmium (Cd) and arsenic (As), through “heavy metal pump” effects during specific growth stages [5]. Furthermore, toxic pyrrolizidine alkaloids (PAs) derived from Asteraceae and Boraginaceae weeds have been detected in commercial tea leaves, posing health risks through soil transfer or direct contamination during harvest [6]. Over 200 weed species coexist with tea plants, predominantly from Poaceae and Asteraceae, followed by Caryophyllaceae, Polygonaceae, Cyperaceae, and Portulacaceae. Dominant species with high adaptability and resistance include *Digitaria sanguinalis*, *Conyza canadensis*, *Stellaria media, Persicaria lapathifolia*, *Cyperus rotundus and Portulaca oleracea* [7].

Due to the drawbacks of manual and physical weed control, current weed management primarily relies on chemical herbicides, applied as pre- or post-emergent treatments through foliar or soil applications. These herbicides target critical physiological processes such as photosynthesis, pigment synthesis, fatty acid metabolism, and amino acid biosynthesis [8]. However, prolonged herbicide use raises environmental concerns, including ecosystem disruption and escalating weed resistance. Consequently, developing eco-friendly alternatives has become imperative [9]. Herein, we address two key questions under the growing demand for sustainable tea production: What factors underpin effective eco-friendly weed control across cultivation systems? And how can these factors be optimized to balance efficacy, yield, and environmental sustainability? This review synthesizes the taxonomic diversity and multidimensional impacts of weeds in tea cultivation systems. It comprehensively reviews the development and application status of both chemical and microbial-based herbicides, with a specific focus on their mechanisms of action. Based on this analysis, novel concepts for herbicide development are proposed, alongside integrated sustainable weed management strategies leveraging chemistry, biotechnology, and precision agriculture principles.

## Ecological diversity and agronomic threats of weeds in tea plantations

### Weed diversity and occurrence patterns in tea plantations

**Fig. 1:**
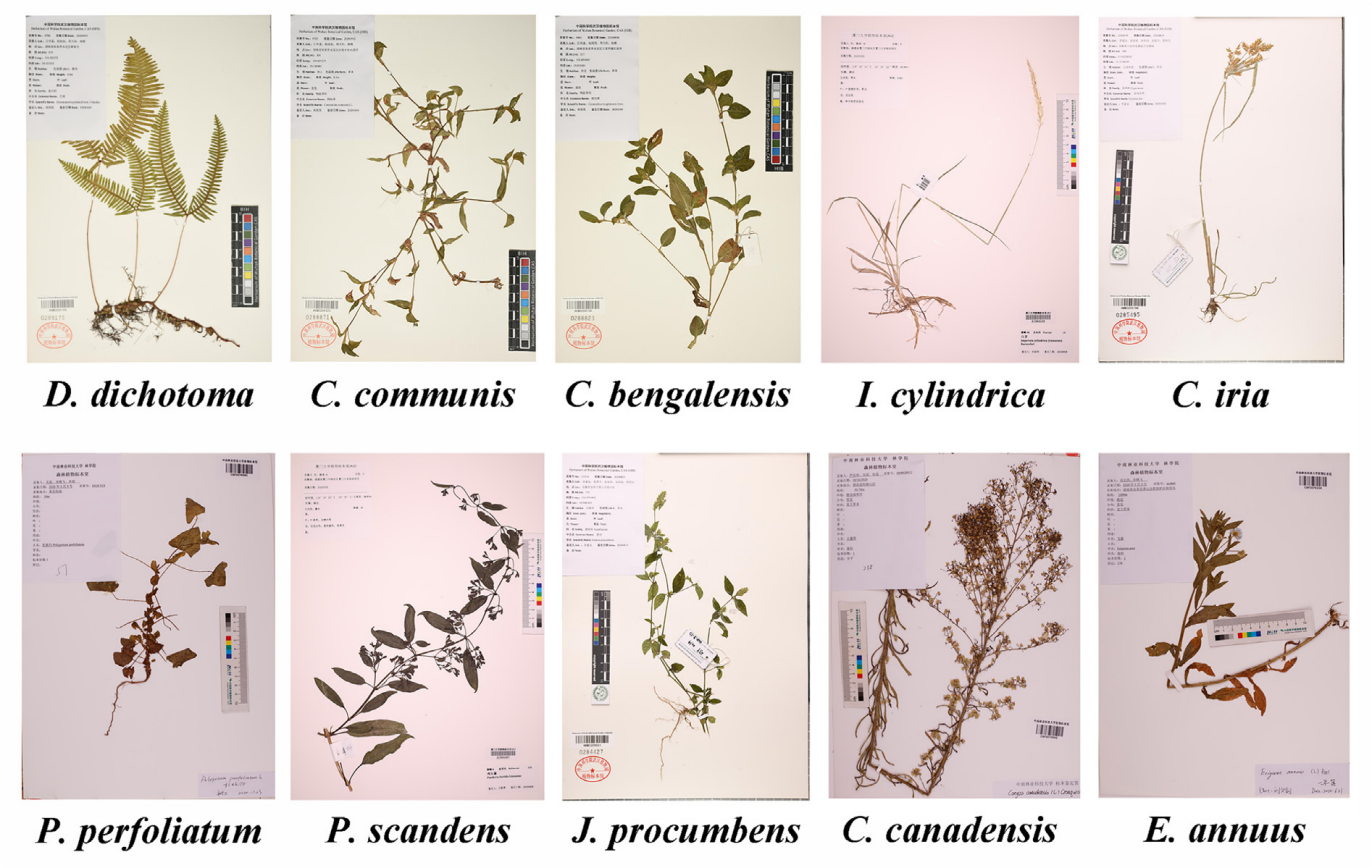
Botanical specimens of severe malignant weeds in tea plantation (http://www.iplant.cn/frps).

A total of 50 weed species from 25 families with significant occurrence rates (frequency > 29 %) have been identified in tea plantations [10,11]. A comprehensive assessment framework is presented to evaluate and classify these weeds based on their ecological impact, reproductive capacity, and management difficulty [12,13]. Ecological competitiveness (40 % weight) is quantified by relative growth rate, canopy coverage, and resource interception efficiency. These metrics collectively indicate a weed's capacity to compete with tea plants for essential resources. Reproductive capacity (35 % weight) is assessed by annual seed production, vegetative propagation potential, and seedbank persistence. High seed output and long-term seedbank viability are key contributors to weed invasiveness and persistence. Management difficulty (25 % weight) is evaluated using herbicide resistance index, mechanical regeneration ability, and labor input requirements. These indicators reflect the challenges involved in controlling specific weed species. Based on the above assessment criteria, weeds are categorized into five hazard tiers: 10 species of severe malignant weeds, characterized by high invasiveness and persistent seedbanks (Table 1, Fig. 1). 15 species of malignant weeds, with moderate competitiveness and rapid dispersal capability. 5 species of general weeds, showing localized distribution and limited ecological impact. 6 species of harmless weeds, exhibiting minimal interference and weak adaptive traits. 14 species of unclassified weeds, for which available data are insufficient for definitive categorization (Supplementary Table S1).

**Table 1:** Classification and ecological traits of dominant malignant weed species in tea plantations.

Family	Species	Life form	Frequency (%)
Gleicheniaceae	*Dicranopteris dichotoma*^★★★^	Perennial herb	5.88
Commelinaceae	*Commelina communis*^★★★^	Annual scattered herb	35.29
	*Commelina bengalensis*^★★★^	Perennial creeping herb	29.41
Poaceae	*Imperata cylindrica*^★★★^	Perennial herb	35.29
Cyperaceae	*Cyperus iria*^★★★^	Annual amphibious herb	41.18
Polygonaceae	*Polygonum perfoliatum*^★★★^	Annual herb	29.41
Rubiaceae	*Paederia scandens*^★★★^	Perennial rattan shrub	11.76
Acanthaceae	*Justicia procumbens*^★★★^	Annual creeping herb	29.41
Asteraceae	*Conyza canadensis*^★★★^	Annual herb	47.06
	*Erigeron annuus*^★★★^	Annual or biennial herb	47.06

Note: ^★★★^ indicates severe malignant weeds. Occurrence frequency (%) is defined as the ratio of a weed’s documented appearances across seventy referenced studies to the total number of surveyed literature sources, multiplied by 100%. This metric provides a quantitative measure of weed prevalence within the surveyed area.

### Integrated impacts of weed infestation in tea ecosystems

#### Multidimensional resource competition in tea agroecosystems

**Fig. 2:**
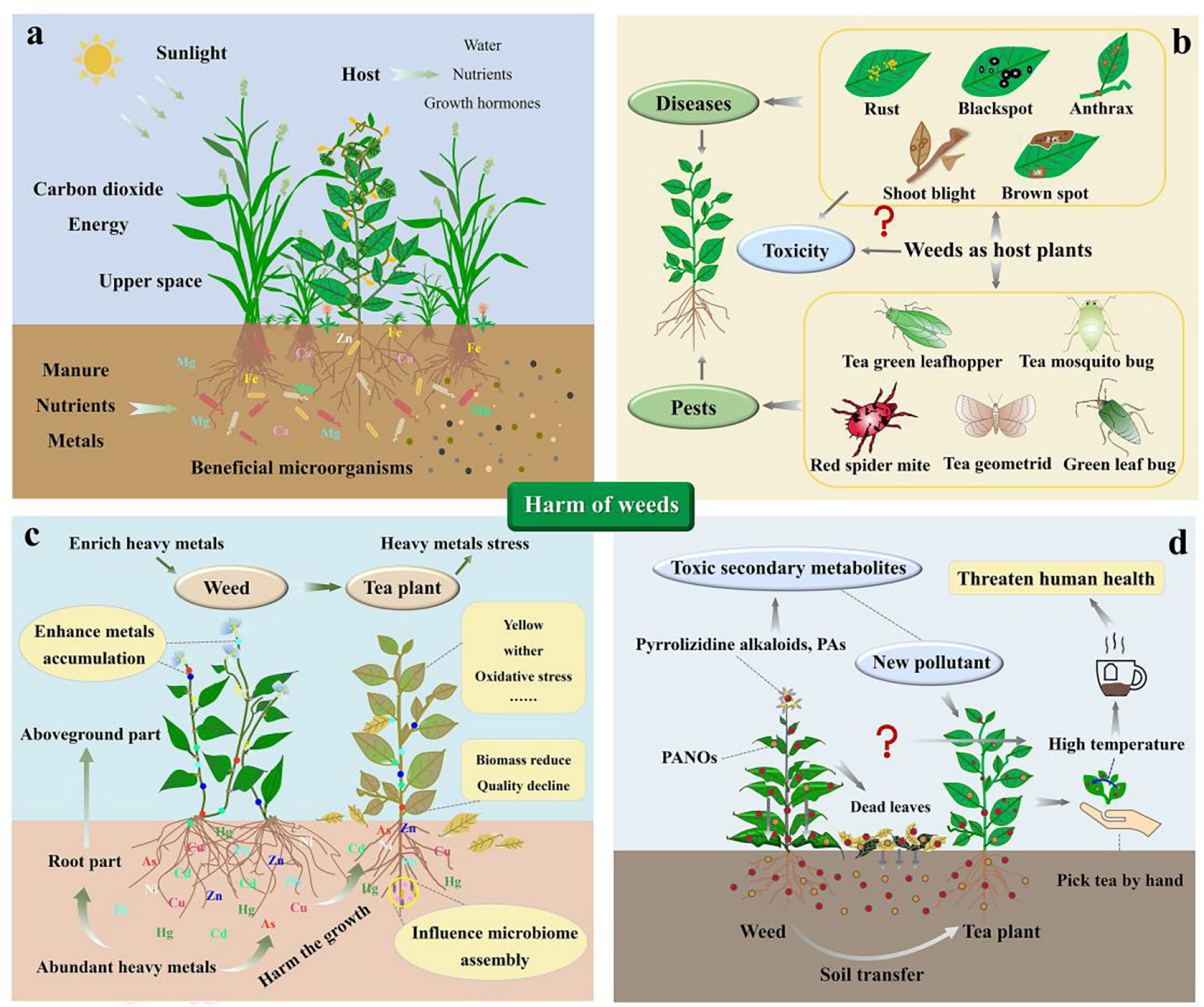
Multifaceted weed-*Camellia sinensis* ecological interactions.

Weeds impose significant competitive pressure on *Camellia sinensis* through multidimensional resource competition, including light interception, water consumption, and nutrient depletion (Fig. 2a). Biotic constraints in tea ecosystems involve height-stratified competition and obligate parasitism. Tall weeds (> 80 cm; e.g., *P. lapathifolium*) reduce canopy light interception and airflow, thereby impairing photosynthesis [14]. Belowground, taproot weeds (e.g., *C. segetum*) access deep soil resources, while stoloniferous species (e.g., *E. indica*) monopolize surface nutrients, leading to nutrient deficiency [15]. Perennial creepers deplete rhizosphere nutrients through regenerative propagules, particularly during the autumn–winter period. Parasitic species (e.g., *Cuscuta chinensis*) form haustoria to extract resources from *C. sinensis*, causing physiological stress, with some maintaining metabolism via arbuscular mycorrhizal symbiosis [[16], [17], [18]].

#### Weeds as reservoirs of tea pathogens and pests

As critical reservoirs for both phytopathogens and arthropod pests, weeds amplify infestation cycles in tea ecosystems, despite supporting certain natural predators (Fig. 2b). Specifically, weeds act as pathobiome reservoirs, sustaining pathogenic fungi through competitive traits such as hyphal expansion and allelochemical synthesis, thereby establishing epidemiological bridges to tea trees [19,20]. For example, *A. alternata* has been isolated from *Ageratum conyzoides*, where it produces the Crofton-weed toxin, indicating that weeds can harbor pathogenic fungi and maintain their activity [21]. Endophytic *Alternaria* spp. have also been detected in *A. conyzoides* leaves, supporting its role as a fungal host [22]. Although direct transmission to tea is unproven, such findings highlight the potential of weeds to sustain inoculum beyond the tea canopy. Shared susceptibility to *Colletotrichum* spp. creates feedback loops: weed proliferation increases inoculum pressure, which elevates tea infection, promotes canopy defoliation, and ultimately favors further weed growth [23,24]. Simultaneously, weeds harbor arthropod pests (e.g., *Apolygus lucorum*), offering oviposition sites and refuge while facilitating phloem-feeding damage to tea [3]. Some weeds (e.g., *Paspalum notatum*) paradoxically exacerbate infestations by providing nutritional subsidies and synchronized microhabitats, despite also supporting predator populations [25].

### Weeds as sources of metal contamination in tea ecosystems

Weeds amplify metalloid risks in tea ecosystems through hyperaccumulation and rhizosphere mobilization, with Pb, Cd, As, and Hg being the predominant contaminants. Species such as *Bidens pilosa* (Cd root sequestration) and *Conyza canadensis* (aerial Cd, Pb, Cu, Zn accumulation) exceed tea tolerance thresholds (Fig. 2c) [26,27]. Microbial synergism enhances metal uptake, e.g., *Pseudomonas*-mediated Cu transport in tea trees [28]. Metalloid stress triggers phytotoxic cascades: Cd induces ROS-mediated oxidative damage, while Pb alters nitrogen–carbon metabolism, affecting caffeine and catechin ratios [29]. Aluminum-hyperaccumulating weeds further compete for Al^3+^, diverting sequestration into vacuoles and impairing xylem function in tea plants [30,31].

#### Weed-derived pyrrolizidine alkaloids: Emerging risks to tea quality and safety

Pyrrolizidine alkaloids (PAs) and their N-oxides (PANOs), toxic secondary metabolites produced by angiosperms, contaminate *Camellia sinensis* products (Fig. 2d). Despite their detection in commercial teas, the ecological drivers and translocation mechanisms remain unclear. Critically, PA bioaccumulation disrupts key quality-related phytochemicals (e.g., polyphenols, amino acids), thereby affecting flavor, antioxidant properties, and sensory attributes [[32], [33], [34]]. Allelopathic transfer occurs at plant–plant interfaces; co-cultivation with *Senecio jacobaea* demonstrates horizontal metabolite exchange with *Jasminum sambac* and *Matricaria chamomilla*, implicating weed–tea rhizosphere interactions as transmission vectors [35]. Field studies reveal multiple contamination pathways: physical incorporation of PA-producing weeds during harvest, with concentrations reaching 90.5 μg/kg [36,37]; edaphic mobilization via root exudates; and phyllospheric deposition of volatile-derived PAs. PA/PANO redox dynamics modulate toxicity: processing oxidizes 68–72 % of PANOs to PAs, whereas brewing preferentially degrades PANOs [38,39]. Global surveys confirm widespread contamination with regional variation [37]. This metabolic plasticity induces tripartite risks involving agronomic (meristem disruption), industrial (processing complications), and public health (chronic dietary exposure) impacts.

a. Competition for light, water, nutrients, space, and influence on rhizosphere microbes and metal dynamics. b. Acting as hosts for major tea diseases and insect pests, increasing infection and infestation risks. c. Accumulation and transfer of heavy metals to tea plants, reducing growth and quality and disrupting microbiomes. d. Release of toxic metabolites such as pyrrolizidine alkaloids, entering tea plants, persisting through processing, and threatening tea safety.

## Chemical weed control in tea plantations: Chemical herbicides application and mechanisms

### Herbicide efficacy and environmental persistence

Chemical herbicides effectively control weeds via non-selective or selective mechanisms, thereby enhancing crop yield [40]. As rapidly evolving agricultural inputs, their operational efficiency and scalability have driven widespread adoption [41]. Modern formulations target key weeds (e.g., *Conyza* spp., *Solanum tuberosum*, *Amaranthus viridis*) by disrupting essential metabolic pathways, including photosynthesis, amino acid biosynthesis, cell division, and carotenoid production [42,43]. Herbicide diversity is categorized based on six parameters: application timing and methods, mechanism of action, target specificity, compound origin, molecular structure, and soil persistence (Table 2).

**Table 2:** Comparative efficacy of chemical herbicides against weed species.

Chemical herbicide	Chemical name	Weed	Usage time	Usage dosage	Activity or Mortality	Application condition & Method	Reference
Glyphosate	N-(phosphonomethyl)glycine	*Amaranthus palmeri*	Seedlings are at 8–10 cm high	Nine doses (0, 31.25, 62.5, 125, 250, 500, 1000, 2000 and 4000 g ae ha^−1^)	Almost no death & Glyphosate-resistance	Laboratory & Spray to plant at 50 cm high	[42]
Glufosinate-ammonium	Ammonium dl-homoalanin-4-(methyl) phosphinate	Annual weeds	Every 3 h (From 06:00 to 24:00)	0.292 kg/ha & Concentration of 15 mg/L	Significant effect & 50 %-60 %	Field & Spray to plant	[50]
Paraquat	1,1-dimethyl-4,4-bipyridine cationic salt	*Bidens pilosa*	Four-leaf stage & six-leaf stage	500 g ha^−1^	Greater than 90 % & greater than 80 %	Laboratory & spray to plant	[44]
		*Ambrosia artemisiifolia*	Preplant Pre-emergence Early post Late post	1120 g ai ha − 1	Significant effect & 90–99 %	Field & spray to plant	[51]

Atrazine	2-chloro-4-ethylamino-6-isopropylamino-1,3,5-triazine	Major broadleaf weeds	Pre-emergence & post-emergence	1160 + 1480 g ai ha^−1^	Significant effect & 80–100 %	Field & combine with s-metolachlor	[45]
Bromoxynil	3,5-dibromo-4-hydroxybenzonitrile	Broadleaf weeds	After seeding and withheld for 24 h	0.56 kg ha^−1^	Greater than 80 %	Greenhouse & spray analogues of bromoxyni	[50]
Metribuzin	4-amino-6-*tert*-butyl-4,5-dihydro-3-methylthio-l,2,4-triazine-5-cyclodextrin	*Fumaria indica*, *Melilotus indica*, *Anagallis arvensis*, and *Phalaris minor*	Two- to four-leaf stage	175 g ai ha^−1^	Significant effect	Field & spray to plant	[52]
Bentazone	3-Isopropy1-1H-2,1,3-benzothiadiazin −4(3H)-one 2,2- dioxide	*Plantago asiatica*, *Agropyron cristatum*, *Digitaria sanguinalis*, et al	Mature (Each year in September)	30 L·ha^−1^	Greater than 73.36 %, even 100 %	Field & spray to plant	[47]
Dicamba	3,6-dichloro-2-methoxy-benzoic acid	Broadleaf weeds	Pre-emergence	101.6–226 ng g soil^−1^	Significant effect & 60–70 %	Field & spray to the soil surface	
Simazine	2,4-Bis(ethylamino)-6-chloro-1,3,5-triazine	*Plantago lanceolata*	4th- to 8th-leaf growth stage	1120 g ai ha^−1^	Significant effect & 78 %	Greenhouse & spray to the leaf surface	[48]
Trifluralin	2,6-dinitro-N	*Chloris virgata*	Spray immediately after sowing	600 g ai ha^−1^	Significant effect	Laboratory & spray to the soil surface	[49]
Imazamox	2-(4,5-dihydro-4-methyl-4-(1-methyl ethyl)-5-oxo-1 h-imidazole-2-yl)-5-(methoxymethyl)-3-pyridine carboxylic acid	Broadleaf weeds	Pre-emergence & post-emergence (three–four-leaf stage)	1124–1244 g ai ha^−1^	High efficacy & Greater than 70 %	Field & Spray at 50 cm high above the plant canopy	[53]
Sulfometuron	2-(3-(4,6-dimethyl pyrimidine-2yl) ureidosulfonyl) benzoic acid	*Lolium multiflorum*	Two- to three-leaf stage	14.5 g ai ha^−1^)	Significant effect & Greater than 90 %	Laboratory & spray to plant	[54]

Chemical herbicides offer broad-spectrum efficacy, low application rates, and reduced mammalian toxicity, yet their environmental persistence poses considerable ecological risks. Volatilization and hydrologic transport via leaching or runoff promote bioaccumulation within ecosystems [44]. Residual phytotoxicity (24–36 months) impairs subsequent crops and disrupts soil carbon and nitrogen metabolism [45]. Intensive usage has led to widespread resistance, primarily via cytochrome P450- and glutathione S-transferase (GST)-mediated detoxification, representing polygenic inheritance. For instance, CYP81A12 and CYP72A31 have been identified *E. phyllopogon*, while GST subtypes such as GSTF1 and GSTU19 are implicated in *C. canadensis* and *L. rigidum*. These enzymes collectively contribute to metabolic resistance in multiple weed species, although the broader molecular signaling networks remain incompletely [46]. Globally, 48 glyphosate-resistant species (e.g., *E. indica*, *C. canadensis*, *L. perenne*) have been documented [47]. Mitigation strategies include physicochemical remediation (e.g., activated carbon adsorption, nanomaterial degradation, membrane filtration) and microbial bioremediation [48]. Notably, rhizosphere-mediated modification of herbicide bioavailability via root exudates represents an emerging resistance mechanism, necessitating innovative management approaches [49].

### Multifaceted mechanisms of chemical herbicide action

**Fig. 3:**
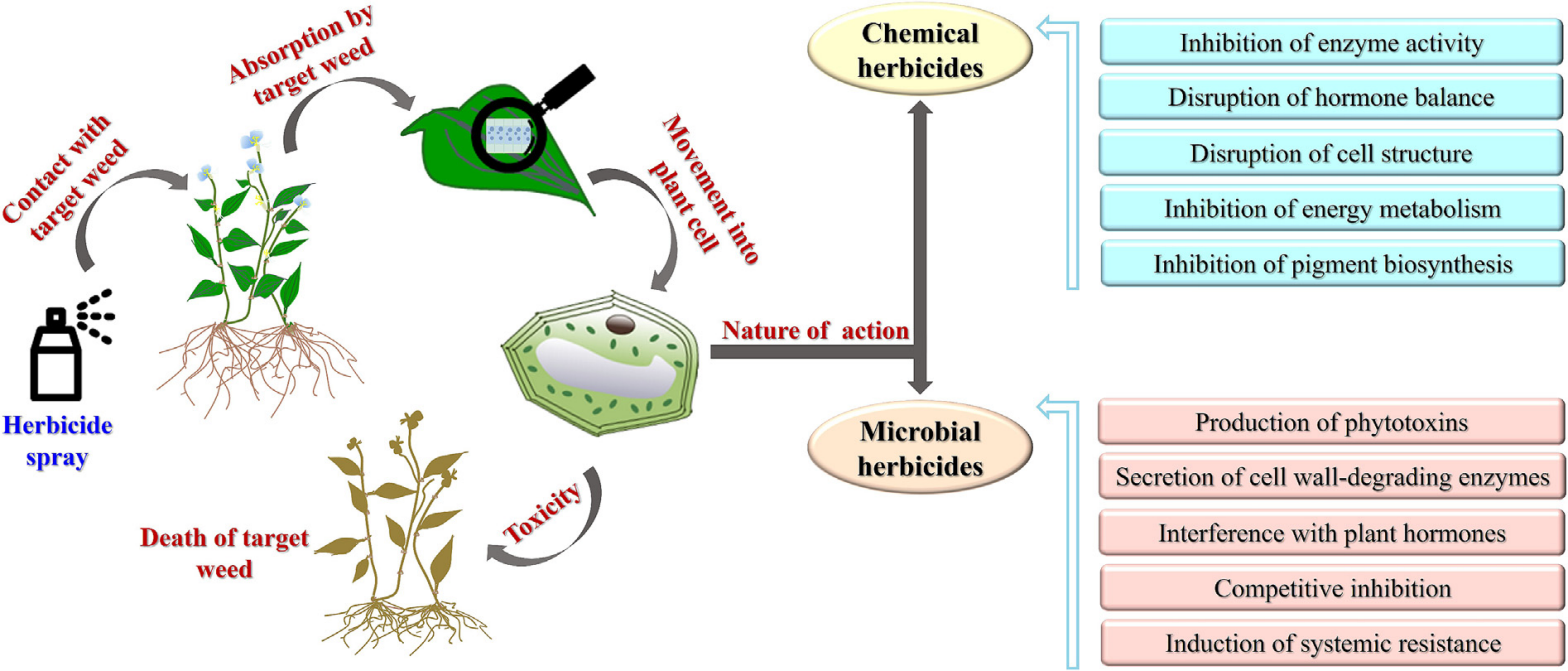
Overview of modes of herbicide actions in weed control agents.

Conventional chemical herbicides act through molecular disruption of key physiological pathways, including enzyme inhibition, hormone imbalance, cell structure damage, energy suppression, and pigment biosynthesis blockage. In contrast, microbial herbicides suppress weeds via ecological and biochemical mechanisms such as phytotoxin production, secretion of cell wall–degrading enzymes, hormonal interference, competitive inhibition, and induction of systemic resistance. Herbicides are absorbed by target weeds, translocated into cells, and ultimately trigger phytotoxic effects leading to weed death, with chemical and microbial agents displaying distinct yet complementary modes of action (Fig. 3) [55,56].

### Inhibition of enzymatic activity

**Fig. 4:**
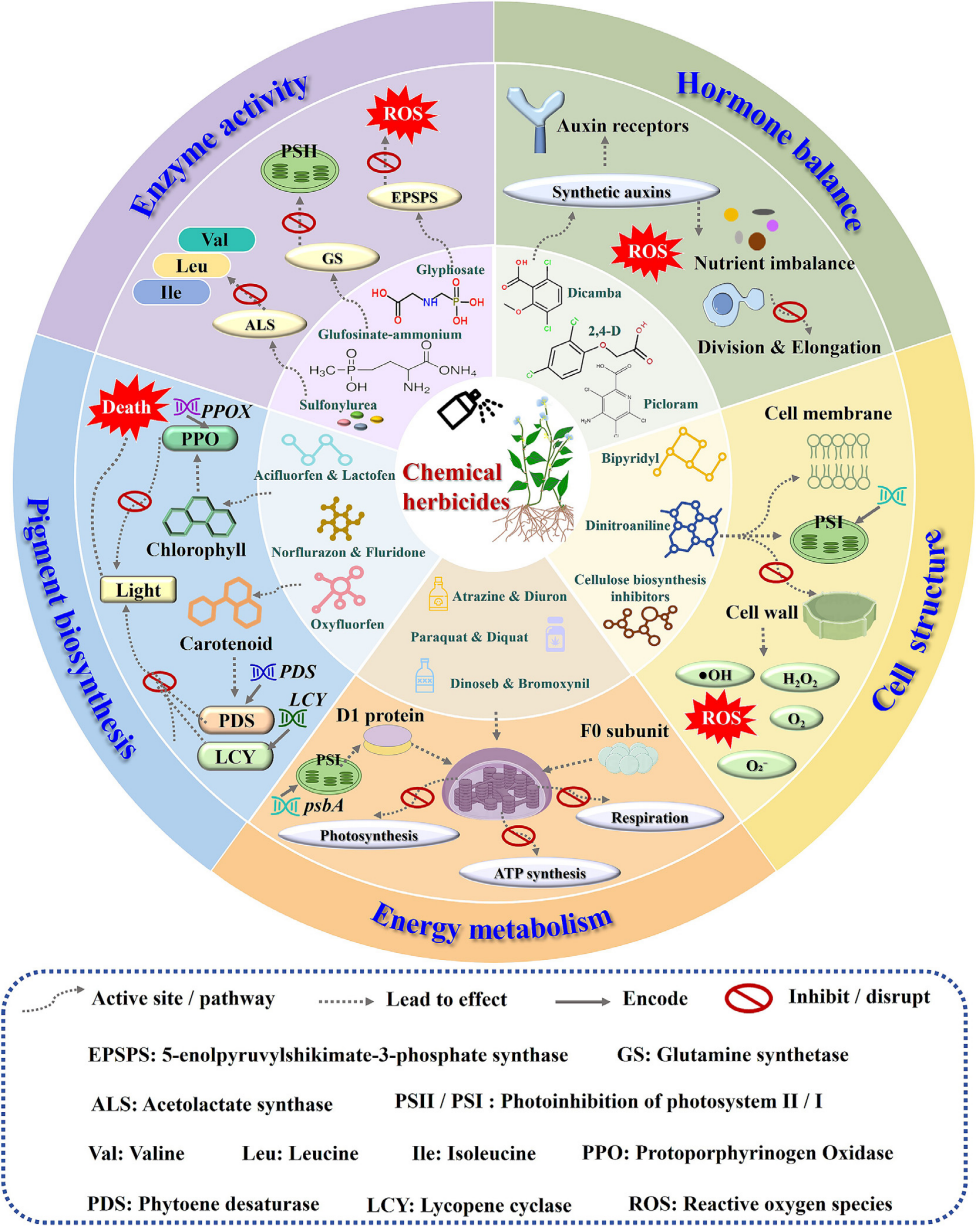
Overview of modes of chemical herbicides action in controlling weed.

Enzymatic inhibition represents one of the most fundamental modes of herbicidal action, directly targeting key metabolic nodes that are indispensable for plant growth and survival. According to the Herbicide Resistance Action Committee (HRAC), glyphosate and glufosinate-ammonium remain the dominant broad-spectrum herbicides applied in global tea plantations, with annual usage estimated at 1,000–1,500 and 500–800 metric tons, respectively. By inhibiting enzymes such as EPSPS in the shikimate pathway, glutamine synthetase in nitrogen assimilation, or acetolactate synthase in branched-chain amino acid biosynthesis, these compounds effectively collapse primary metabolism and trigger systemic plant mortality (Fig. 4).

Glyphosate inhibits 5-enolpyruvylshikimate-3-phosphate synthase (EPSPS), a key enzyme in the shikimate pathway. This plastid-localized synthase catalyzes the condensation of shikimate-3-phosphate (S3P) with phosphoenolpyruvate (PEP) to form EPSP, the committed step in aromatic amino acid biosynthesis, such as tryptophan, tyrosine, phenylalanine [57]. Glyphosate's structural mimicry of PEP enables competitive binding at the enzyme's active site, effectively blocking substrate access and terminating the shikimate pathway [58]. The resulting amino acid deficit induces systemic metabolic collapse, compounded by secondary effects including photorespiratory dysfunction and ROS-mediated oxidative damage [59]. Glufosinate-ammonium operates through distinct enzymatic targeting, primarily inhibiting glutamine synthetase (GS) in the glutamate-ammonia ligase cycle, manifesting as rapid chlorosis and complete necrosis within 120 h post-application [60]. This metalloenzyme normally catalyzes ATP-dependent amination of glutamate to glutamine, essential for ammonia detoxification and nitrogen assimilation [61]. As a phosphinic analog of glutamate, glufosinate competitively occupies GS's substrate-binding pocket, disrupting ammonium incorporation [62]. The consequent NH_3_ accumulation uncouples electron transport chains. Secondary phytotoxicity arises through multiple pathways, such as PSII photoinhibition, thioredoxin system oxidation, and programmed cell death initiation [63]. Field efficacy is enhanced through transgenic crop compatibility-plants expressing bar or pat genes encode phosphinothricin acetyltransferases that acetylate glufosinate's amino group, rendering it non-toxic [64]. Sulfonylurea herbicides exemplify another inhibition strategy, targeting acetolactate synthase (ALS) in branched-chain amino acid synthesis. This FAD-dependent enzyme catalyzes the first common step in valine, leucine and isoleucine biosynthesis through parallel decarboxylation-condensation reactions [65]. Herbicide binding at the ALS catalytic pocket induces allosteric distortion, preventing substrate orientation for catalysis. The resulting amino acid starvation halts mitosis through cyclin-dependent kinase suppression, while accumulated α-ketoacid precursors induce membrane peroxidation and chloroplast disintegration [66].

### Disruption of hormone balance

Another major strategy of chemical herbicides is the disruption of endogenous hormone balance, particularly through the use of auxin-mimetic compounds. Synthetic auxin herbicides, including 2,4-D, dicamba, and picloram, structurally resemble indole-3-acetic acid (IAA) and competitively bind to auxin receptor complexes, thereby hijacking the SCF ubiquitin ligase signaling pathway. This results in abnormal activation of auxin response cascades, leading to uncontrolled cell elongation, nutrient imbalance, and oxidative stress [67]. Such interference causes severe developmental abnormalities and ultimately plant death, with broadleaf weeds being especially susceptible due to their auxin sensitivity (Fig. 4).

Synthetic auxin herbicides (SAHs) exemplified by 2,4-dichlorophenoxyacetic acid (2,4-D), dicamba, and picloram exert phytotoxicity through molecular mimicry of endogenous indole-3-acetic acid (IAA). These compounds competitively bind to TIR1 and AFB auxin receptor complexes in the SCF ubiquitin ligase system, triggering constitutive activation of auxin response factors (ARFs). This aberrant signaling cascade induces primary phytotoxic effects, such as hyperactivation of expansins and xyloglucan endotransglucosylase causing uncontrolled cell elongation, and induction of ACC synthase-mediated ethylene overproduction [68]. Simultaneously, they lead to a cascade of secondary effects, like oxidative stress, nutrient imbalance, and senescence and death. In addition, synthetic auxin herbicides are selective, meaning they target broadleaf weeds while sparing grasses and certain crops. This selectivity is due to differences in auxin metabolism and receptor sensitivity between plant species [69]. Emerging resistance mechanisms challenge SAH efficacy, the widespread use of these herbicides has led to the evolution of resistance in some weed populations, some weeds can rapidly detoxify synthetic auxins, rendering them ineffective [70]. Structural variations among SAHs play a key role in determining field performance. Dicamba's methyl ether moiety enhances membrane permeability and enables control of auxin-transport–resistant biotypes. In contrast, picloram's pyridine ring system facilitates xylem translocation, which supports suppression of deep-rooted perennials [56,71]. Currently, resistance of them is less common but can occur through enhanced metabolism or target-site mutations, particularly in weeds that have developed metabolic detoxification mechanisms.

### Disruption of cell structure

Chemical herbicides can also directly disrupt plant cellular architecture, compromising membranes, microtubules, and cell walls that are essential for structural integrity and physiological function. Bipyridyl herbicides such as paraquat and diquat generate reactive oxygen species (ROS) via photosystem I, initiating oxidative membrane damage and programmed cell death. Dinitroaniline compounds interfere with spindle formation by binding tubulin, whereas cellulose biosynthesis inhibitors weaken cell walls by blocking cellulose synthase activity. Collectively, these effects undermine cell integrity and result in irreversible tissue collapse (Fig. 4).

Bipyridyl herbicides generate reactive oxygen species (ROS) in the presence of light. Such as paraquat and diquat, are reduced by photosystem I (PSI) in chloroplasts to form a paraquat radical (PQ^+^). This radical reacts with molecular oxygen (O_2_) to produce superoxide radicals (O_2_^−^), which are highly reactive and initiate a cascade of ROS, including hydrogen peroxide (H_2_O_2_) and hydroxyl radicals (OH•) [72,73]. During this process, ROS activate mitogen-activated protein kinase (MAPK) pathways, which regulate stress responses and programmed cell death (PCD). It found *psbA* gene encodes the D1 protein of PSI, which is critical for paraquat's action, and mutations in *psbA* can confer resistance to paraquat by reducing its binding affinity [74,75]. Dinitroaniline herbicides (e.g., trifluralin and pendimethalin), bind to α- and β-tubulin, the building blocks of microtubules, preventing their polymerization, this disrupts spindle formation during mitosis, leading to abnormal cell division [76,77]. However, mutations in the *TUB* genes, which encode tubulin proteins, can confer resistance to dinitroaniline herbicides [78]. In addition, Cellulose biosynthesis inhibitors (e.g., isoxaben and indaziflam), inhibit cellulose synthase (CESA) enzymes, which are encoded by the CESA gene family, leads to weakened cell walls and impaired cell elongation [79,80].

### Inhibition of energy metabolism

A further critical target of herbicides lies in energy metabolism, where photosynthesis, respiration, and ATP synthesis form the core of cellular energy production. Photosystem II inhibitors such as atrazine and diuron block electron transport, leading to ROS accumulation and chloroplast damage, while mitochondrial inhibitors like dinoseb uncouple electron transport from ATP synthesis. Other compounds directly inhibit ATP synthase, halting proton-driven phosphorylation. The cumulative effect of these disruptions is profound energy depletion, rendering plants unable to sustain growth or survival (Fig. 4).

Herbicides like atrazine and diuron bind to the D1 protein (encoded by the *psbA* gene) in PSII, blocking electron transfer from water to plastoquinone. This disrupts the photosynthetic electron transport chain (PETC), leading to reactive oxygen species (ROS) accumulation and chloroplast membrane damage [81,82]. As research has found atrazine resistance in *Amaranthus tuberculatus* is linked to *psbA* mutations and overexpression of detoxifying enzymes like glutathione S-transferases (GSTs) [83]. Like paraquat and diquat, reduced by PSI to form radicals that react with oxygen, generating reactive oxygen species (ROS), causing lipid peroxidation and energy depletion [84]. Meanwhile, Herbicides that inhibit respiration disrupt the process that plants generate ATP through the oxidation of organic molecules, then leading to energy depletion [85]. Like dinoseb and bromoxynil, disrupt the proton gradient across the mitochondrial membrane, uncoupling electron transport from ATP synthesis. This leads to energy dissipation as heat [86]. Like rotenone and cyanide, inhibit complex I or IV of the mitochondrial electron transport chain, preventing ATP synthesis [87,88]. In addition, herbicides that inhibit ATP synthesis directly disrupt cellular energy levels. Like oligomycin and venturicidin, bind to the F_0_ subunit of ATP synthase, blocking proton flow and ATP production [89,90].

### Inhibition of pigment biosynthesis

Inhibition of pigment biosynthesis represents a distinct herbicidal mechanism that compromises both photosynthetic capacity and photoprotection. Herbicides targeting chlorophyll biosynthesis, such as PPO inhibitors, induce accumulation of phototoxic intermediates that trigger oxidative membrane damage under light. Similarly, carotenoid biosynthesis inhibitors like norflurazon and fluridone disrupt light-harvesting and ROS scavenging, resulting in photobleaching and oxidative stress [91]. By dismantling pigment-dependent energy capture and protection systems, these herbicides cause lethal damage to plant photosynthetic machinery (Fig. 4).

Chlorophyll is essential for capturing light energy during photosynthesis. Herbicides that inhibit chlorophyll biosynthesis disrupt this process, leading to photobleaching and energy depletion. Acifluorfen and lactofen inhibit protoporphyrinogen oxidase (PPO), an enzyme involved in the synthesis of chlorophyll and heme. Inhibition leads to the accumulation of protoporphyrin IX, which generates singlet oxygen (^1^O_2_) in the presence of light, causing lipid peroxidation and membrane damage [92,93]. And the *PPOX* gene encodes PPO, mutations in this gene can confer resistance to PPO inhibitors by reducing herbicide binding affinity [94]. Another type is glutamate-1-semialdehyde aminotransferase (GSAT) inhibitors such as oxyfluorfen. These compounds inhibit GSAT, an enzyme required for the synthesis of 5-aminolevulinic acid (ALA), a chlorophyll precursor, resulting in chlorophyll deficiency and photobleaching. And its resistance is determined by the *GSAT* gene [95,96]. Similarly, carotenoids are also essential for photoprotection and light harvesting. Herbicides that inhibit carotenoid biosynthesis disrupt these functions, leading to photobleaching and oxidative stress [97]. Norflurazon and fluridone inhibit phytoene desaturase (PDS), an enzyme involved in the synthesis of carotenoids, leads to the accumulation of phytoene, causing chlorophyll degradation and photobleaching [98]. So, the *PDS* gene encodes PDS to express resistance. There are also herbicides like amitrole, which are inhibitors of lycopene cyclase (LCY), an enzyme involved in the synthesis of carotenoids. It leads to the accumulation of lycopene, causing chlorophyll degradation and photobleaching. The *LCY* gene encodes LCY, which is crucial for developing resistance to mutations [99,100]. In addition, herbicides can cause a series of secondary effects while inhibiting pigment biosynthesis, typically involving oxidative stress and programmed cell death (PCD). This is because the accumulation of phototoxic intermediates protoporphyrin IX and the degradation of chlorophyll lead to the generation of ROS, and then ROS-induced damage triggers PCD pathways [75,101].

## Biological weed control in tea plantations: Bioherbicide application and mechanisms

Biological weed control employs living organisms and their bioactive products as environmentally sustainable alternatives. It integrates three modalities: (1) Phytogenic herbicides using allelopathic extracts to disrupt weed germination and growth; (2) Zoogenic agents deploying herbivores or animal biomolecules for targeted suppression; (3) Microbial formulations harnessing pathogens to synthesize phytotoxic metabolites. This phyto-zoo-microbial synergy provides a multifunctional herbicide alternative, mitigating agrochemical pollution while enhancing biodiversity conservation.

### Plant-derived bioherbicides

**Fig. 5:**
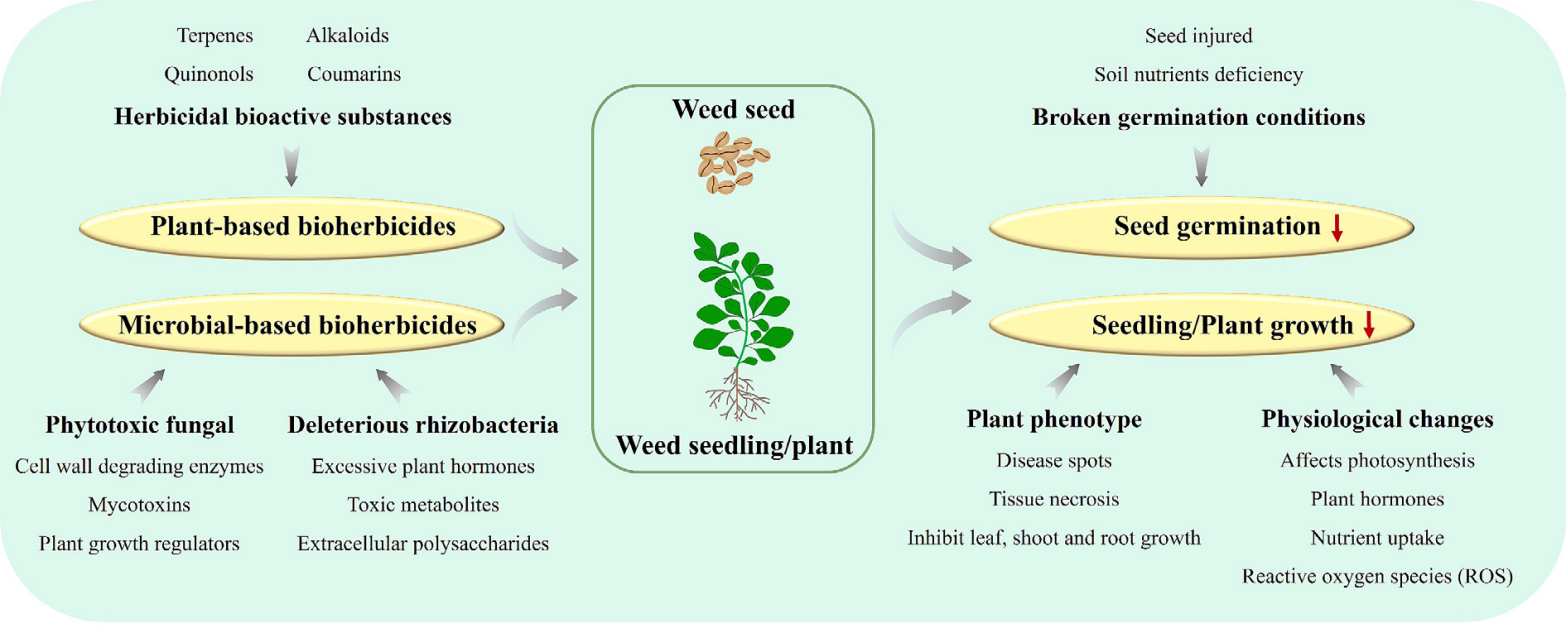
Schematic representation of the modes of action of phytogenic and microbial bioherbicides in weed suppression.

Phytogenic bioherbicides represent a major subclass of bioherbicides, utilizing phytotoxic compounds extracted from botanical sources. Over 30 plant families have yielded more than 100 bioactive molecules with validated weed-suppressive activity [54]. These agents exhibit multi-target phytotoxicity through five primary mechanisms: inhibition of seed germination, suppression of seedling growth, disruption of photosynthesis, interference with hormone regulation, and impairment of nutrient uptake (Fig. 5, Table 3). Key allelopathic compounds—including terpenoids, alkaloids, quinones, and coumarins—are released via root exudation or foliar leaching, inducing dose-dependent phytotoxic effects on plant developmental processes [102]. Their species-specific activity ensures compatibility with crops and vertebrates while retaining strong herbicidal efficacy.

**Table 3:** Comparative efficacy of phytogenic bioherbicides against weed species.

Plant Herbicidal active substances	Phytogenic bioherbicide types	Targeting weeds	Against effect (%) Mode of action in weeds	Reference
*Artemisia argyi* Caffeic acid, schaftoside	Water-soluble extract	Six weeds (*Brassica pekinensis*, et al)	Significant against effect Inhibit dicotyledons and monocotyledons by seed germination and seedling growth.	[103]
*Trigonella foenum-graecum* Monosubstituted trioxazonane	Root extract	*Orobanche crenata*	27 %-54 % Inhibit seed germination.	[104]
*Medicago sativa* –	Water extract	*Elymus nutans*	Significant against effect Inhibit seed germination rate, seedling length, root length, and dry weight.	[105]
*Cynara cardunculus* Naringenin, myricitrin, and quercetin	Plant extract	*Trifolium incarnatum*	Significant against effect Inhibit seedling, roots, and hypocotyl growth, induce oxidative stress.	[106]
*Sapindus mukorossi* Hederagenin 3-o-β-d-xylopyranosyl-(1–3)-α-l-rhamnopyranosyl-(1–2)-α-l-arabinopyranoside	Pulp ethanol extract	*Trifolium pratense*	Significant against effect Strong inhibition of root growth and elongation.	[107]
*Albizia richardiana* 3-(2-hydroxyethyl)-2,4,4-trimethyl-2cyclohexen-1-one	Plant aqueous extract	*Lactuca sativa*, *Lolium multiflorum*	Almost completely inhibited Inhibit shoot and root with increasing extract concentration.	[108]
*Lavandula* spp. (seven species) Coumarin, 7-methoxycoumarin	Stem and leaf extract, flower extract	*Lolium rigidum*	Significant against effect Inhibit root growth, even a concentration of 10 % almost completely limited the growth.	[109]
*Euphorbia heterophylla* Terpenoids	Essential oil	*Cenchrus echinatus*	93.95 %, 84.6 %, and 57.8 % Inhibit seed germination, root, and shoot growth.	[110]
*Triticum aestivum* P-coumaric acid, propionic acid	Shoot aqueous extract	*Lolium rigidum*	12 %-100 % Inhibit seed germination and root growth.	[111]
*Semenovia transiliensis* Furanocoumarin compounds	Shoot extract	*Lactuca sativa*, *Agrostis stolonifera*	Moderate against activity Inhibit the growth.	[112]
*Verbena incompta* Verbascoside	Plant extract	Lotus japonicus, et al	Significant against effect Inhibit the root growth.	[113]
*Cymbopogon citratus*, *Eucalyptus cladocalyx* Five components	Essential Oil	*Sinapis avensis*	Significant against effect Inhibit seed germination and seedling growth.	[114]
*Piper betel* Phenylpropanoid	Leaf extract	Four weeds (*Chloris barbata*, et al)	Significant against effect Inhibit seed germination and seedling growth, especially the roots.	[115]
*Melilotus officinalis* Dihydrocoumarin	Plant extract	*Echinochloa crus-galli*	Inhibit seedling growth by oxidative stress (disrupt the cell membrane, and reduce the root cell activity, et al).	[116]
*Oryza sativa* Ergosterol peroxide, 7-oxo-stigmasterol	Root extract	*Echinochloa crus-galli*	Significant against effect Inhibit coleoptile growth, shoot, and root lengths.	[117]
*Artemisia argyi* –	Water extract	*Oryza sativa*	Significant against effect Destroy the root and leaf by reactive oxygen species homeostasis, and accumulation of soluble sugar and chlorophyll synthesis.	[118]
*Chromolaena odorata* –	Leaf extract	*Echinochloa crus-galli*, *Amaranthus viridis*	Significant against effect Inhibit photosynthesis process, aggravate lipid peroxidation and cell disruption.	[119]
*Sicyos angulatus* Phenolic compounds	Seed extract	*Lactuca sativa*	20 %–23 % Inhibit plant growth of photosynthesis, regulating phytohormones and nutritional elements.	[120]

### Insect-derived bioherbicides

Zoogenic bioherbicides represent a subclass of bioherbicides that employ host-specific phytophagous insects as targeted agents for weed biological control. Such strategies require rigorous screening criteria, including strict host specificity, ecological plasticity, and elevated reproductive capacity. *Coleopteran* species dominate current biocontrol applications, with taxonomic prevalence descending through Curculionidae, Chrysomelidae, Lepidoptera, Pyralidae, Diptera, Hymenoptera, Orthoptera and Thysanoptera [121]. In tea plantations, insect-derived bioherbicides have so far received limited attention, yet preliminary observations suggest their potential value.

Recent advances in insect-derived bioherbicides highlight both the taxonomic diversity of candidate agents and the innovation of their control mechanisms. Although systematic studies within tea plantations remain limited, insights from broader agroecosystems provide valuable references for potential application. For example, *Rhinoncomimus latipes* and *Diorhabda carinolata* have demonstrated high host specificity in suppressing *Persicaria perfoliata* and *Tamarix* spp., underscoring the feasibility of Curculionidae and Chrysomelidae as large-scale biocontrol agents [122]. Similarly, *Listronotus setosipennis* and *Galerucella placida* exhibit selective feeding on *Parthenium hysterophorus* and Polygonaceae weeds (*Rumex dentatus*, *Polygonum glabrum*), respectively [123,124]; while studies on *Herpetogramma basalis* suggest the potential of Lepidopteran insects for sustained suppression of *Alternanthera philoxeroides* [125]. Collectively, these representative cases offer important ecological references for identifying candidate insects to manage dominant weeds in tea gardens, such as *Ageratum conyzoides* and *Bidens pilosa*. Beyond direct herbivory, innovative mechanisms have also been reported. For instance, *Carmenta mimosa* induces branch necrosis in *Mimosa pigra* through pheromone signaling [126]; synergistic feeding interactions between *Zygogramma bicolorata* and *Epiblema strenuana*, as well as stem parasitism of *Delairea odorata* by *Parafreutreta regalis*, markedly enhance control efficacy [127]. Even oviposition-mediated disruption, as in *Contarinia nasturtii* on cruciferous weeds, reveals unconventional suppression pathways [128]. Despite these advances, operational challenges persist in balancing ecological safety with control efficiency, particularly regarding non-target effects and adaptive management of insect-plant interactions.

### Microbial-derived bioherbicides

The escalating prevalence of herbicide-resistant weed biotypes, driven by intensive synthetic agrochemical use, poses critical challenges to agricultural sustainability and ecosystem integrity. In response to these dual pressures of weed resistance evolution and environmental constraints on conventional herbicide development, microbial bioherbicides represent a distinct subclass of bioherbicides and have emerged as a frontier solution. These biopesticides exploit phytotoxic compounds from microbial metabolism as structural templates for novel herbicide design, offering targeted weed suppression while mitigating ecological collateral damage [129]. Current research identifies two principal derivation pathways for microbial herbicidal agents: viable biocontrol inoculants comprising plant-pathogenic fungi, bacteria, actinomycetes, and viral particles, with fungal and bacterial formulations dominating field applications; and secondary metabolic derivatives biosynthesized through microbial fermentation, including cyclic peptides, diterpenoid phytotoxins, polyketide macrolides, and phenolic derivatives. Herein, we mainly illustrate the following two categories:

(1) Fungal-based microbial bioherbicides

Phytotoxic fungi and their secondary metabolites represent one of the most promising microbial herbicide alternatives for weed control. During their interaction with weeds, these fungi secrete pathogenic factors primarily comprising cell wall-degrading enzymes, mycotoxins, and phytohormones (Fig. 5, Table 4). The plant cell wall constitutes the primary physical barrier against pathogen invasion, which pathogenic fungi overcome through the production of enzymatic complexes including pectinases, chitinases, cellulases, and proteases. These enzymes synergistically degrade the cuticle and cell wall components, facilitating fungal colonization and systemic invasion of weed tissues [130]. A notable example is *Fusarium graminearum*, which secretes cellulases, xylanases, and pectinases during infection, effectively decomposing structural polysaccharides in host cell walls to enable hyphal penetration and tissue maceration [131]. Beyond enzymatic degradation, emerging research highlights the critical role of *Fusarium*-derived mycotoxins such as fusaproliferin, beauvericin, enniatins, and moniliformin in enhancing pathogen virulence during infection processes [132]. The phytopathogenic invasion mechanism involves a sophisticated interplay between enzymatic action and toxin-mediated pathogenesis. These fungal metabolites compromise plant defenses through multiple pathways, inducing necrotic lesions, disrupting cellular metabolism, impairing membrane integrity, and ultimately triggering programmed cell death [133]. *Alternaria* and *Nimbya* species exemplify this strategy through production of alternariol and monomethylalternariol, which inhibit chloroplast electron transport to suppress plant growth [134]. Mitochondrial and peroxisomal dysfunction induced by *Ustilago maydis* infection not only impairs fungal development but also generates cytotoxic short-chain fatty acids that accelerate weed cell death [135]. In addition, the pathogenic arsenal further extends to microbial manipulation of plant hormonal networks. Numerous fungal species synthesize phytohormone analogs including auxins, cytokinins, ethylene, gibberellins, abscisic acid (ABA), brassinosteroids, and bioactive oligosaccharides during host colonization [136]. This hormonal interference critically modulates weed-pathogen interactions, as evidenced by *Magnaporthe oryzae*'s production of indoleacetic acid to suppress plant defense responses [137]. Conversely, exogenous ABA has been shown to stimulate *Botrytis cinerea* virulence by enhancing hyphal growth and appressorium formation [138]. Such multifaceted attack strategies underscore the complex biochemical warfare underlying fungal-weed interactions, where coordinated enzymatic, toxicological, and hormonal mechanisms collectively overcome plant resistance mechanisms.

(2) Bacterial-based microbial bioherbicides

**Table 4:** Compendium of phytopathogenic fungi and bacteria exhibiting herbicidal activity against target weeds.

Strain	Herbicidal active substances	Targeting weeds	Mode of action in weeds	Reference
Endophytic fungal	Plant toxins	*Senna occidentalis*, *Ipomoea grandifolia*	Inhibit seed germination, photosynthesis, and plant growth.	[151]
*Alternaria*	AF-toxins, ACT-toxins, and AAL-toxin, et al	N/A	Inhibit in vitro development of calli, pollen, roots, and shoots.	[152]
*Cochliobolus australiensis*	Radicinin (dihydropyranopyran-4,5-dione)	*Cenchrus ciliaris*	Toxicity and partial leaf tissues show necrosis.	[153]
*Cochliobolus australiensis*	(±)-3-deoxyradicinin (a synthetic analogue of radicinin)	*Solanum lycopersicum*	Aggravate leaves chlorosis, ion leakage, hydrogen peroxide production, and membrane lipid peroxidation.	[154]
*Colletotrichum gloeosporioides* BWH-1	Dirhamnolipid	Nine weeds including *Echinochloa crusgalli*, *Alopecurus aequalis*	Inhibit root length and fresh weight.	[155]
*Pyricularia grisea*	Radicinin (dihydropyranopyran-4,5-dione)	*Cenchrus ciliaris*	Show high toxicity and larger necrotic lesions.	[156]
*Penicillium* and *Aspergillus*	Citrinin	*Ageratina adenophora*	Inhibition of chlorophyll synthesis causes leaf lesions.	[157]
*Trichoderma koningiopsis*	Cellulase and lipase	*Euphorbia heterophylla*	Cause leaf necrosis and shrinkage.	[158]
*Bipolaris bicolor* SYNJC-2–2	N/A	*Eleusine indica*	Hyphae infect leaves and cause cell death and necrotic lesions.	[159]
*Fusarium oxysporum*	N/A	*Orobanche* spp.	Cause ROS damage, and degrades protein metabolism and synthesis, resulting in apoptosis.	[160]
*Pseudomonas ogarae* F113	Phloroglucinol compounds	*Phelipanche ramose*, *Orobanche cumana*	Inhibit seed germination rate and reduce root length.	[161]
*Streptomyces* strain-329	Metabolites	Grass and broadleaf weeds	Increase leaf electrolytic leakage and MDA production, inhibit plant growth.	[162]
*Bacillus inaquosorum* NL1, *B. safensis* NL2	Phenolics, n-alkanes	*Cenchrus echinatus*	Phytotoxic effects against seed germination with 100 % inhibition rate.	[163]
*Bacillus wiedmannii*	Herbicidal proteins	*Avena fatua*, *Lolium perenne*, et al*.*	Inhibit fresh and dry weight of stems and roots.	[164]
*Xanthomonas campestris*	N/A	*Ranunculus asiaticus*	Causing leaf blight on a buttercup with necrotic lesions and leaf yellowing.	[165]
*Pseudomonas*	Cyanide	*Echinochola crus-galli*, *Hordeum murinum*, et al.	Significantly inhibit plant growth.	[166]
Deleterious rhizobacteria	Volatiles, diffusible metabolites	*Amaranthus palmeri*	Inhibit seed germination in vitro and plant development.	[167]
*Rathayibacter toxicus*	Corynetoxin	*Lolium perenne*	Destruct plant tissue.	[168]
*Pantoea*	N/A	*Digitaria ischaemum*	Inhibit seed germination, destroy root hairs, and even plant death.	[169]
*Bacillus altitudinis* D30202	4-hydroxy-3-methoxy cinnamic acid, two indole derivatives	*Avena fatua*	Inhibit plant growth, influence yield and quality.	[170]

Note: N/A indicates that no relevant information is currently available in the literature.

Bacteria exhibiting herbicidal potential predominantly originate from rhizosphere microbial communities associated with weed-infested soils, with their modes of action (Fig. 5, Table 4). Notable examples include *Xanthomonas* and *Pseudomonas* species capable of inducing characteristic leaf spot lesions and vascular wilt in target weeds. Designated as deleterious rhizobacteria (DRB), these microbial agents, encompassing *P. putida*, *Stenotrophomonas maltophilia*, and *Enterobacter taylor*, demonstrate efficient colonization on weed seed surfaces, root tissues, and rhizosphere soil matrices. Their establishment disrupts critical developmental processes through multiple mechanisms, suppressing seed germination viability, impairing radicle emergence, and arresting seedling establishment [139]. The phytotoxic effects of DRB are principally mediated through biosynthesis of bioactive metabolites. Volatile compounds including hydrogen cyanide and dimethyl disulfide synergize with non-volatile phytotoxins to induce cellular damage, while overproduction of phytohormones and extracellular polysaccharides exacerbates physiological stress in weeds [140]. Experimental evidence demonstrates this dual-action strategy, *P. fluorescens* strain G2-11 significantly inhibits both germination and vegetative growth of *Setaria viridis*, while wheat rhizosphere-derived bacterial suspensions induce chlorosis and mortality in *Avena fatua* through foliar application [141,142]. Specific DRB strains employ specialized biochemical weapons, such as *P. fluorescens* isolates from *Raphanus raphanistrum* rhizospheres that secrete root-inhibitory hydrogen cyanide [143]. Nutrient competition and metabolic interference constitute additional suppression mechanisms. *Pseudomonas* and *Enterobacter* species from *Trifolium repens*-associated microbiomes disrupt nitrogen fixation through rhizotoxic metabolite production, effectively stunting seedling development [144]. The regulatory capacity of *Enterobacter* I-3 extends to hormonal modulation, suppressing gibberellin biosynthesis while enhancing abscisic acid synthesis to block germination initiation [145]. Volatile-mediated phytotoxicity is exemplified by *Bacillus cereus* strains whose ammonia emissions reduce *Lactuca sativa* germination rates and promote seedling necrosis [146,147]. Structural analogs of plant metabolites further contribute to growth inhibition, as evidenced by 5-aminolevulinic acid production in *Brassica juncea*-inhibitory rhizobacteria [148]. Hormonal dysregulation represents a sophisticated attack strategy. Exogenous indole-3-acetic acid (IAA) interferes with α-amylase activity to disrupt seed reserve mobilization, while bacterial-synthesized phenolic compounds like p-coumaric acid impair photosynthetic efficiency through dual inhibition of chlorophyll biosynthesis and light-harvesting complex expression [149,150]. These multipronged biochemical interactions underscore the complex ecophysiological interplay between DRB and their target weeds, where coordinated metabolic assaults overcome plant defense systems through simultaneous disruption of developmental and physiological processes.

### Multifaceted mechanisms of microbial herbicide action

**Fig. 6:**
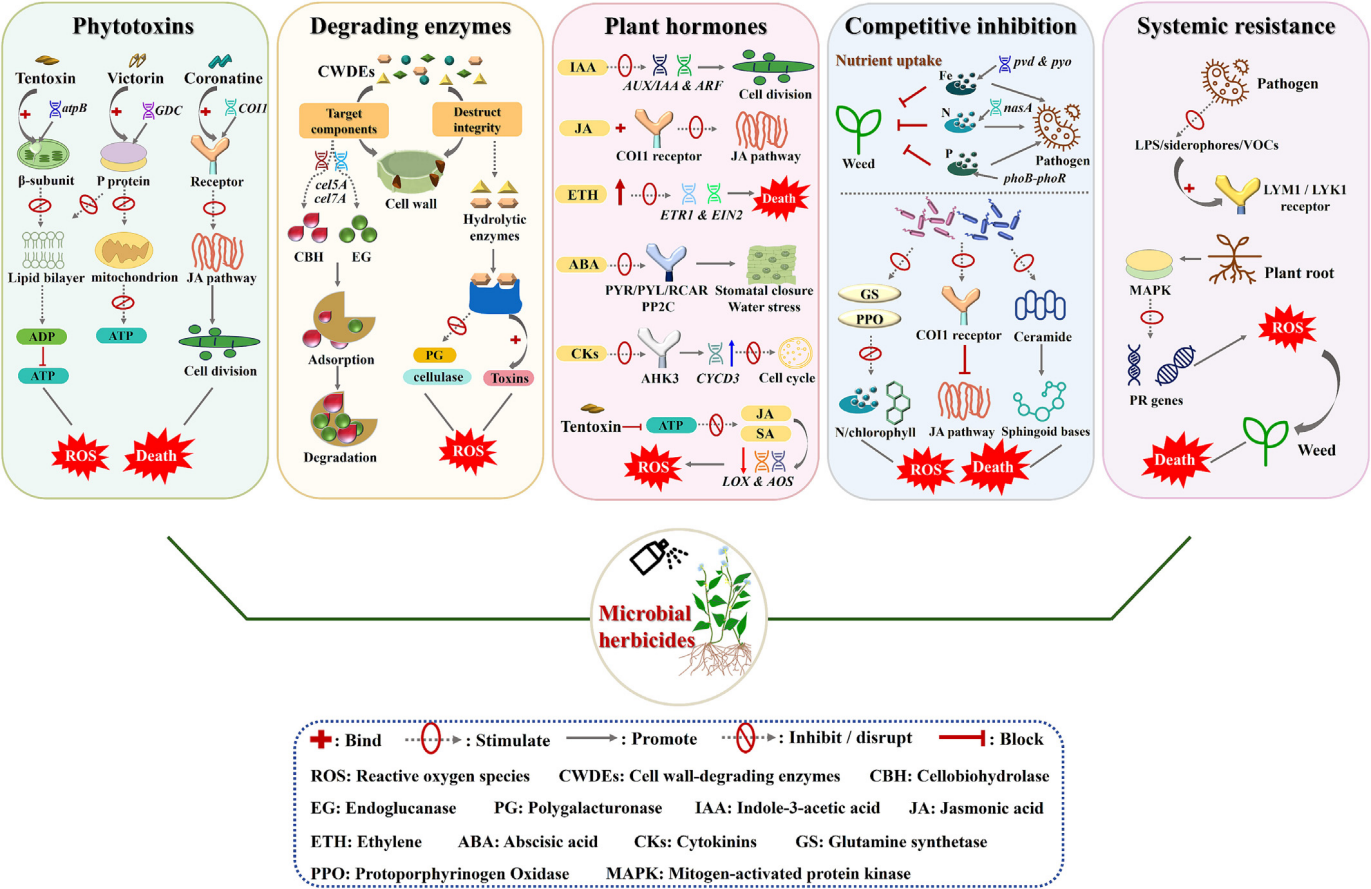
Overview of modes of microbial herbicides action in controlling weeds.

Microbial herbicides suppress weeds through ecological interactions and biomolecular warfare. Their strategies include the biosynthesis of phytotoxic secondary metabolites, secretion of cell wall-degrading enzyme complexes, phytohormone mimicry to disrupt developmental signaling, rhizospheric niche competition, and induction of systemic acquired resistance in host plants (Fig. 6) [171,172].

### Production of phytotoxins

Microbial herbicides derived from bacteria, fungi, or other microorganisms often exert their herbicidal effects by producing bioactive toxins that disrupt essential physiological processes in plants [173]. These toxins disrupt cell membranes, impair energy metabolism, interfere with signaling pathways, and induce oxidative stress, ultimately causing growth inhibition and plant death (Fig. 8).

Many microbial herbicides produce toxins that directly disrupt the lipid bilayer of cell membranes, leading to leakage of cellular contents and loss of membrane potential. Like *Alternaria alternata* produces tentoxin, a cyclic peptide that binds to the chloroplast ATP synthase and blocks ATP synthesis, thereby inhibiting photosynthesis and compromising membrane integrity [174,175]. Moreover, some microorganisms also produce specific toxins targeting beneficial crops. *Cochliobolus victoriae* produces victorin toxin, which targets the glycine decarboxylase complex in oats, disrupting mitochondrial function and membrane stability [176]. Meanwhile, some microbial toxins specifically target membrane proteins, such as ion channels and transporters, disrupting cellular homeostasis. *Pseudomonas syringae* produces syringomycin, binds to membrane lipids and forms ion channels, the formation of ion channels leads to the leakage of potassium (K^+^) and other ions [177]. The strain also can produce coronatine, activate the jasmonic acid signaling pathway by binding to the COI1 receptor, encoded by the *COI1* gene, leading to uncontrolled cell division and growth [178]. In addition, several microbial toxins promote the accumulation of reactive oxygen species (ROS). For instance, *A. alternata* produces alternariol, which impairs mitochondria and triggers ROS generation [179]. Likewise, cercosporin generates ROS by absorbing light energy and transferring it to oxygen, producing singlet oxygen and other reactive species [180].

### Secretion of cell wall-degrading enzymes

Microbial herbicides derived from bacteria, fungi, and actinomycetes often secrete cell wall-degrading enzymes (CWDEs) to breach plant structural defenses. These enzymes hydrolyze key polysaccharides such as cellulose, pectin, chitin, and hemicellulose, leading to tissue maceration, nutrient leakage, and ultimately plant death (Fig. 6).

Representative examples illustrate their modes of action. *Colletotrichum gloeosporioides* secretes endoglucanases (EGs) and cellobiohydrolases (CBHs), which degrade cellulose microfibrils and compromise cell wall strength [181]. Some studies have also found that the expression of cellulase genes *cel5A* and *cel7A* in *Trichoderma reesei* is activated by the transcription factor XYR1 under carbon starvation [182]. Besides, Pectin is a complex polysaccharide, and degradation of pectin disrupts cell adhesion and wall integrity [183]. *B. subtilis* produces the pectate lyase PEL3, which disrupts the middle lamella of Chenopodium album; loss of this enzyme markedly reduces herbicidal efficacy. Moreover, pectin fragments released during enzymatic degradation function as damage-associated molecular patterns (DAMPs), activating plant immune responses [184,185]. Other enzymes also contribute to wall degradation. Chitinases cleave chitin-like components, while xylanases hydrolyze hemicellulose. For instance, *Streptomyces scabies* produces Xyn11A, which synergizes with cellulases to enhance wall breakdown in *Arabidopsis* [186,187]. Moreover, CWDEs are frequently encoded in gene clusters and secreted through specialized systems, as in *P. fluorescens*, where the *gsp* cluster controls PG and cellulase secretion [188]. Importantly, CWDEs often act synergistically with microbial toxins, such as the combination of pectinases and alternariol in *A. alternata*, which amplifies phytotoxicity by suppressing host ROS-scavenging enzymes [189].

### Interference with plant hormones

Plant hormones, such as auxins, ethylene, abscisic acid, cytokinin, jasmonic acid and salicylic acid, regulate various aspects of plant growth and development [190,191]. Microbial herbicides interfere with these hormonal pathways by producing mimics, inhibitors, or modulators that disrupt hormonal balance, resulting in abnormal growth, tissue damage, and ultimately plant death (Fig. 6).

Auxin signaling is a frequent microbial target. *Agrobacterium tumefaciens* produces indole-3-acetic acid (IAA) and cytokinin mimics, which induce gall formation by stimulating uncontrolled cell division. Meanwhile, IAA and cytokinin mimics activate auxin-responsive genes, such as *AUX/IAA* and *ARF*, leading to uncontrolled cell division and tumor formation [192]. *Pseudomonas syringae* produces coronatine, a toxin that mimics jasmonic acid (JA) and interferes with auxin signaling. In the process, Coronatine binds to the COI1 receptor, encoded by the *COI1* gene, activating JA signaling and suppressing auxin responses [193]. Microbial herbicides also manipulate ethylene levels to disrupt plant growth. Such as *Pseudomonas savastanoi* produces ethylene, activates the *ETR1* and *EIN2* genes, which regulate senescence and abscission, which accelerates senescence and tissue decay [194]. Some fungi secrete ABA mimics to induce premature stomatal closure. Such as *Botrytis cinerea* and *Fusarium* spp. produces ABA during infection, activating host ABA receptors (PYR/PYL/RCAR) and PP2C phosphatases, leading to the constitutive activation of ABA signaling. This cause stomatal closure and water stress, causing wilting and plant death [195,196]. Besides, pathogenic microbes may overproduce cytokinins (CKs) to disrupt source-sink relationships. Such as *Rhodococcus fascians* synthesizes methylated CKs via the fas operon, causing abnormal shoot proliferation in *Taraxacum officinale*. That is CK receptors (AHK3) are also constitutively activated, upregulating *ARR* response regulators and disrupting cell cycle gene *CYCD3* [197]. Additionally, microbial effectors often suppress JA and salicylic acid (SA) cross-talk disruption to weaken plant immunity. Such as *A. alternata* produces tentoxin, which inhibits chloroplast ATP synthase, reducing JA and SA biosynthesis. This suppresses *LOX* and *AOS* gene expression, rendering *Abutilon theophrasti* susceptible to oxidative stress [152,198].

### Competitive inhibition

Microbial herbicides often exert their herbicidal effects through competitive inhibition, a process where microbial compounds compete with essential substrates or cofactors in plant metabolic pathways. This competition interferes with nutrient acquisition, enzyme activity, hormone perception, and secondary metabolism, ultimately leading to nutrient deprivation, metabolic collapse, and plant death (Fig. 6).

Competition for nutrients is a common strategy [199]. Such as certain rhizosphere bacteria compete with weeds for iron ions by producing siderophores, and iron deficiency leads to hindered photosynthesis and respiration in weeds. *P. fluorescens* is a very typical example, its siderophores bind to iron with high affinity, encoded by the *pvd* and *pyo* gene clusters, depriving plants of this essential nutrient, leading to chlorosis and growth inhibition [200]. It has found that microbial strains such as *P. fluorescens* and *B. subtilis* employ high-affinity transporters to outcompete weeds for ammonium (NH_4_^+^) and nitrate (NO_3_^−^). Meanwhile, *B. subtilis* expresses *nasA* to convert NO_3_^−^ into NH_4_^+^, depleting soil nitrate levels and suppressing weed root development [201,202]. Phosphate-solubilizing microbes (PSMs) like *P. putida* and *Penicillium oxalicum* secrete organic acids to solubilize inorganic P, while simultaneously monopolizing it via high-affinity transporters. Under P limitation, it activates the *phoB-phoR* two-component system, inducing alkaline phosphatases and P transporters [203,204]. Microbial herbicides can produce compounds that competitively inhibit key enzymes in plant metabolic pathways. Such as *Streptomyces* spp. produce phosphinothricin, a competitive inhibitor of glutamine synthetase (GS) that involved in nitrogen assimilation, preventing the conversion of glutamate to glutamine [205]. There are also studies that have found *Phoma macrostoma* produces macrocidins, an enzyme involved in chlorophyll and heme biosynthesis. It binds to the active site of PPO, encoded by the PPOX gene, preventing the conversion of protoporphyrinogen to protoporphyrin IX [205]. Besides, microbial herbicides can produce compounds that competitively inhibit hormone receptors or signaling components. Such as *P. syringae* produces coronatine, a toxin that mimics JA and competitively inhibits the COI1 receptor, encoded by the *COI1* gene [193]. *Fusarium* spp. produce fumonisins that inhibit ceramide synthase, causing sphingolipid imbalance, oxidative stress, and cell death [206].

### Induction of systemic resistance

Microbial herbicides can act not only through direct toxicity but also by indirectly enhancing crop defenses via induced systemic resistance (ISR). This strategy involves microbial recognition by plant pattern recognition receptors (PRRs), activation of defense-related signaling pathways, and priming of defense responses (Fig. 6).

ISR is mediated by rhizosphere microorganisms such as *P. fluorescens*, *Bacillus* spp., and mycorrhizal fungi. These organisms secrete elicitors including lipopolysaccharides, siderophores, and volatile organic compounds (VOCs) that prime host defense without directly harming weeds [207,208]. Reported that *P. fluorescens* produces LPS, which bind to PRRs like LYM1/LYK1 in *Arabidopsis*. This interaction activates mitogen-activated protein kinase (MAPK) cascades, leading to defense gene expression [209,210]. It also found that pyoverdine siderophores from *Pseudomonas* spp. chelate iron, indirectly inducing ISR by modulating root exudate composition and enhancing plant iron uptake [211]. Besides, ISR differs from systemic acquired resistance (SAR) by relying on JA and ET signaling rather than SA pathways [212]. *P. fluorescens* strain secretes pseudobactin siderophores that activate *Arabidopsis* ISR via the MYC2 transcription factor in the JA pathway. Enhanced root lignification and reduced *Amaranthus retroflexus* root penetration [213]. *B. amyloliquefaciens* GB03 releases volatile organic compounds (VOCs) like acetoin, which upregulate *MYC2* and *ERF1* in *Arabidopsis*, priming JA/ET defenses. Suppressed growth of *Chenopodium album* through allelochemical cross-talk [214,215]. And some microbes combine ISR with direct weed suppression [216]. Such as *Phoma* spp. secrete phytotoxins and upregulate host phenylalanine ammonia-lyase (PAL) for lignin synthesis, blocking weed growth [217]. In addition, the initiation of defense responses is also crucial in the key molecular mechanisms of ISR. The NPR1 (non-expressor of pathogenesis-related genes, PR genes) protein is central to ISR. It can translocate to the nucleus upon redox changes, interacting with TGA transcription factors to activate *PR* genes [[218], [219], [220]]. It is also worth noting that ISR primes NADPH oxidases (RBOHD/F) to amplify ROS bursts during subsequent stress, strengthening cell wall lignification and weed suppression [221,222].

## Main steps of current herbicide development: From screening to spraying

**Fig. 7:**
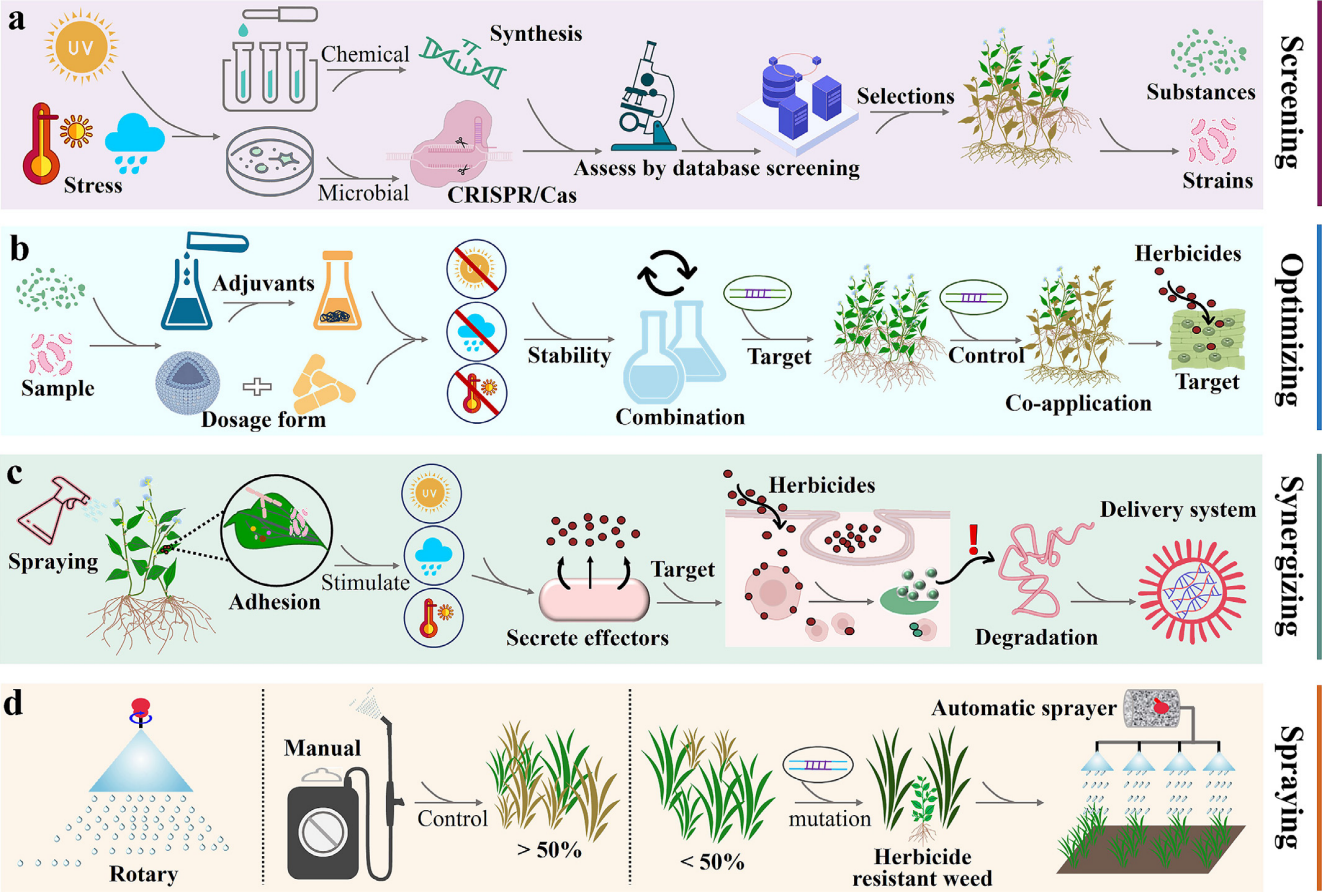
Main steps in current herbicide development a. Active ingredient screening pipeline. b. Formulation optimization strategies. c. Advanced delivery systems. d. Traditional herbicide application method.

Despite their fundamentally distinct origins and modes of action, chemical and microbial herbicides are undergoing remarkable technological convergence in their developmental trajectories. In response to the escalating challenges of herbicide resistance and environmental sustainability, this section presents a comprehensive analysis of four pivotal pathways that are reshaping both fields: (1) novel active ingredient discovery, (2) formulation optimization, (3) advanced delivery systems, and (4) application technologies (Fig. 7).

**Fig. 8:**
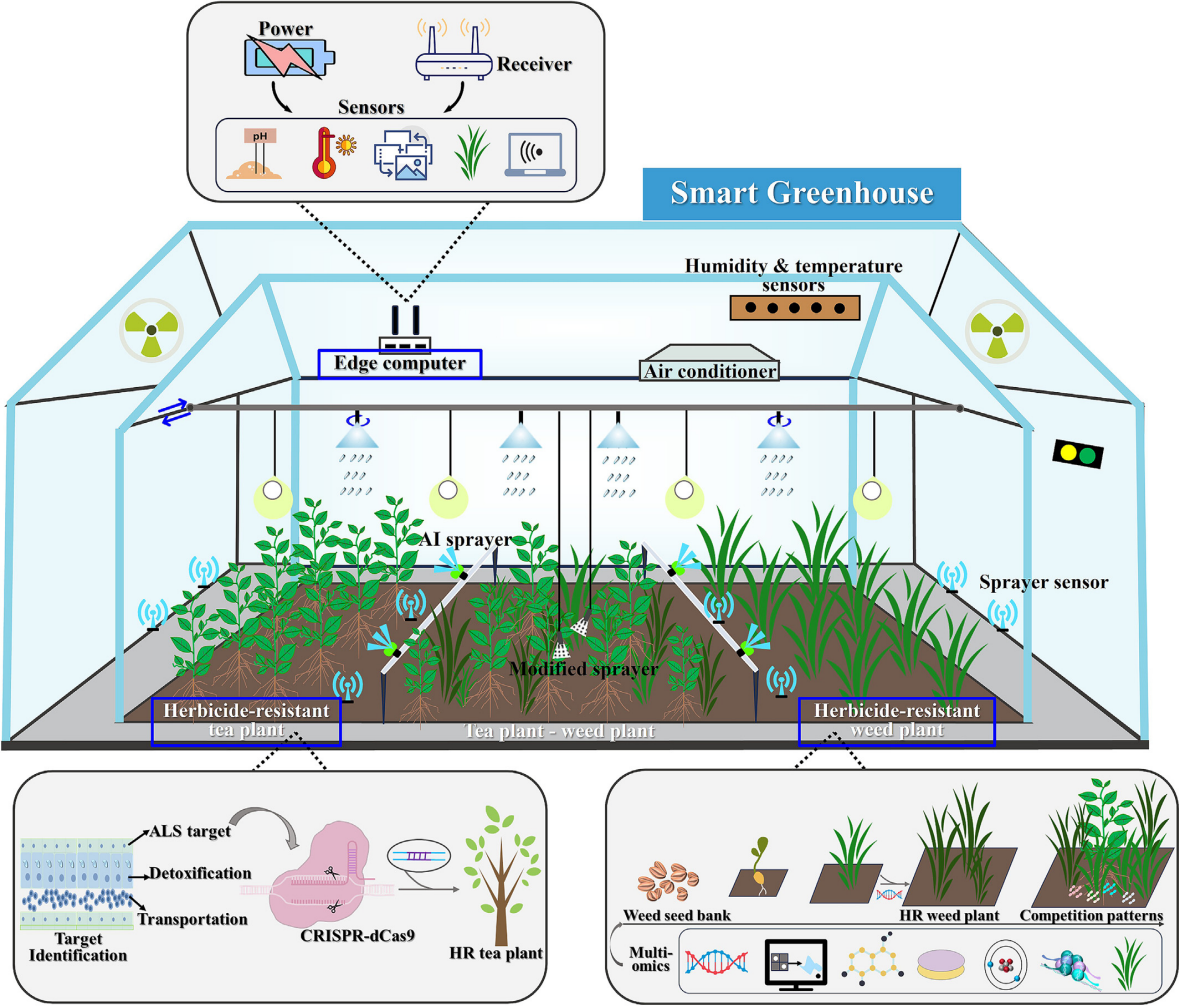
Strategies for dosage reduction and efficacy enhancement of herbicides.

### Screening the herbicide active ingredients

Microbial herbicides, which utilize naturally occurring or genetically modified microorganisms to control weeds, offer a promising alternative to traditional chemical herbicides. Screening involves stress-induced metabolite discovery, microbial strain selection, CRISPR/Cas-based engineering, and chemical database mining, together providing diverse candidate compounds. Herein, we review cutting-edge strategies in (1) mining and genetically modifying high-efficacy microbial herbicide strains and (2) redesigning chemical herbicides through biosynthesis and degradation engineering (Fig. 7a).

Once promising microbial candidates or biosynthetic gene clusters (BGCs) are identified, CRISPR-Cas genome editing plays a pivotal role in strain optimization. Key engineering strategies include enhancing pathogenicity and herbicidal activity through knockout of self-protection genes or overexpression of virulence factors, as demonstrated in *Colletotrichum truncatum*, where multiplex CRISPR-Cas9 disruption of detoxifying enzyme genes accelerated host kill kinetics and reduced sporulation requirements [223]. Expanding host range can be achieved by engineering strains to produce multiple host-specific toxins or modifying host recognition factors, such as adhesins or effectors, to minimize non-target effects [224]. Synthetic biology has enabled the refactoring of phytotoxin BGCs, exemplified by the thaxtomin cluster from *Streptomyces* scabies, which was heterologously expressed in *S. coelicolor* with promoter optimization to boost production tenfold [225]. Additionally, improving environmental fitness through traits like desiccation tolerance, UV resistance, or enhanced root colonization ensures field persistence, as seen in *Pseudomonas fluorescens* engineered for osmolyte synthesis, which improved survival in clay-based formulations under drought stress [226]. Parallel advances in biosynthesis engineering have enabled the redesign of chemical herbicides for improved efficacy and reduced environmental impact, leveraging synthetic biology to develop novel modes of action targeting specific weed pathways. The Atrazine degradation pathway has been optimized for enhanced efficiency. Meanwhile, microbial consortia, such as *Variovorax*, *Comamonas testosteroni*, and *Hyphomicrobium sulfonivorans* in linuron degradation highlight the potential of community-based bioremediation, with metabolic modeling further enabling the design of synthetic microbiomes for targeted herbicide degradation in soil [227].

### Optimizing the dosage form and add efficient additives

Optimizing herbicide formulations and incorporating advanced adjuvants significantly enhance environmental stability, targeted delivery efficiency, and resistance management potential. This review systematically analyzes three critical strategies: (1) Formulation engineering through nanocapsules or granular systems to improve active ingredient stability and site-specific targeting; (2) Functional adjuvants including UV blockers and penetration enhancers potentiate herbicide performance in complex field environments; (3) Microbial-chemical hybrids delay resistance evolution via synergistic multi-target mechanisms (Fig. 7b).

Recent advances in herbicide formulation technologies demonstrate significant improvements in efficacy and environmental sustainability. Nanocapsules effectively encapsulate active ingredients, providing protection against environmental degradation while enhancing tissue penetration efficiency [228]. Complementary granular systems enable controlled herbicide release, extending field activity by 30–50 % and reducing application frequency [229]. These advanced delivery systems collectively enhance target specificity while minimizing off-target effects and environmental contamination. Adjuvant innovations further optimize performance, with UV-blocking additives reducing photodegradation by up to 70 % and surfactant-based penetration enhancers increasing foliar uptake by 40–60 % [230]. Particularly promising are synthetic herbicides that synergistically combat resistance evolution. *Cercospora rodmanil* and 2,4-D combinations demonstrate 49 % greater efficacy than conventional herbicides alone, while *Ascochyta caulina* metabolite complexes enhance *Chenopodium album* control by 32 % compared to chemical-only treatments. These integrated approaches not only expand weed control spectra but also reduce chemical load by 35–45 %, representing a significant stride toward sustainable weed management [231,232]. We propose that the microbial-chemical synergy paradigm warrants expanded exploration.

### Synergizing with stabilized delivery systems

Herein, we comprehensively analyze how advanced nanocarriers, microencapsulation, and stimulus-responsive platforms enhance herbicide delivery by improving precision, stability, and ecological compatibility, thereby mitigating environmental persistence and weed resistance. These systems integrate spraying, adhesion, and effector secretion into protective carriers that ensure targeted release (Fig. 7c).

Nanocarrier technologies for chemical herbicides have achieved relative maturity. Ligand-modified nanoparticles preferentially accumulate in weed cuticles, reducing spray drift by 30–60 % compared to conventional formulations, while polymeric nanocapsules decrease atrazine leaching by 89 %, thus mitigating groundwater contamination [233,234]. Microencapsulation provides spatiotemporal control via tunable release kinetics, exemplified by cellulose acetate microcapsules, which reduce dinitroaniline volatilization by 70 % [235]. Nanoformulations concurrently enhance active ingredient stability: mesoporous silica nanoparticles extend the UV half-life of glyphosate by 3.8-fold via molecular shielding; lipid–polymer hybrids improve Amaranthus hybridus systemic translocation by 40 % through optimized stomatal uptake; and PLGA-based encapsulation sustains field activity for over 120 days [236,237]. Stimuli-responsive systems further minimize non-target effects: pH-sensitive chitosan/alginate nanoparticles selectively release dicamba in the weed rhizosphere (pH 5.5–6.0), curtailing soil microbial disruption by 63 %; enzyme-triggered nanoherbicides targeting *Lolium rigidum* β-glucosidases reduce the effective dosage by 75 %, thereby delaying resistance [[238], [239], [240]]. In contrast, microbial herbicide research lags but shows significant potential. Encapsulation enhances bioherbicide viability, with chitosan–alginate beads extending *Pseudomonas fluorescens* field persistence from 3 to 28 days via UV protection, and lyoprotectant nanocapsules boosting *Colletotrichum truncatum* spore storage survival to 90 % at 4 °C [238]. Nonetheless, critical gaps persist in field-scale stability, urging the development of cryoprotective nanomatrices and pathogen–host-specific ligands. Engineered phage-mediated RNAi delivery also represents an underexplored frontier.

### Broadcast herbicide spraying

Traditional herbicide application methods, primarily relying on uniform spraying techniques, exhibit substantial inefficiencies that compromise agricultural sustainability. Manual and rotary spraying often cause overuse and drift, while automatic sprayers improve precision and reduce waste. These limitations manifest in two critical ways: (1) over-application, resulting in increased production costs, accelerated evolution of herbicide-resistant weeds, and environmental contamination; and (2) under-application, leading to inadequate weed control and subsequent yield losses (Fig. 7d).

Broadcast spraying frequently deposits 30–60 % of herbicides outside target areas due to spray drift, with only a fraction reaching the intended weed species. This imprecision necessitates higher application rates, increasing costs by 20–40 % while exacerbating resistance selection pressure [241]. For instance, repeated glyphosate overuse has led to resistance in 58 weed species globally, with *Amaranthus palmeri* populations showing 10- to 100-fold resistance increases in major cropping systems [242]. Environmentally, excessive herbicide inputs contaminate soil and water systems—atrazine, for example, persists in groundwater at concentrations exceeding 3 μg/L in intensive agricultural regions, threatening aquatic ecosystems [243]. Conversely, suboptimal dosing fails to control weeds during critical growth stages. Field studies demonstrate that 15–30 % reductions in recommended herbicide rates enable weed escapes, causing 5–15 % yield losses in cereals and up to 40 % in broadleaf crops [244]. This is particularly problematic for late-emerging weeds that evade early-season sprays yet compete aggressively during crop reproductive stages. Moreover, conventional sprayers cannot differentiate weeds from crops, wasting herbicides on non-target vegetation.

## Future perspectives

Despite significant progress in sustainable weed management, tea plantations continue to face critical challenges in maintaining ecosystem health. While microbial herbicides offer a promising alternative, they are currently unable to replace chemical herbicides completely as the dominant weed control strategy. Instead, the most viable path forward involves parallel advancements: optimizing microbial herbicide efficacy while simultaneously mitigating the persistent limitations of chemical herbicides. Both chemical and microbial herbicides share fundamental limitations, particularly in active ingredient development, resistance evolution, and field application challenges. Herein, we focus on strategies for dosage reduction and efficacy enhancement, offering innovative solutions through integrated weed, tea and application perspectives.

### Multi-omics approaches: Deciphering weed resistance strategies

As “super environmental stress tolerators,” weeds possess unique growth characteristics and remarkable adaptability, rapidly evolving herbicide resistance that demands stage-specific management strategies. Although crop genomics has advanced substantially, critical knowledge gaps remain for resistant weed species, with reference genomes still lacking for key species like *C. communis* and *D. sanguinalis* [245]. In particular, urgent priorities include sequencing the genomes of *D. sanguinalis*, *C. canadensis*, *L. rigidum*, *A. palmeri*, and *E. crus-galli*, which represent major herbicide-resistant weeds of global and regional significance. Multi-omics approaches are proving essential for deciphering resistance mechanisms: transcriptomics reveals herbicide-induced expression patterns, as demonstrated by CYP450 and GST upregulation in glyphosate-resistant *E. crus-galli* [246]; metabolomics identifies detoxification pathways, such as the RrCYP72A15-mediated mesotrione resistance in *R. raphanistrum* [247]; and proteomics uncovers molecular adaptations, including elevated CYP450s and GSTs in resistant biotypes [248]. However, these single-omics approaches alone cannot fully elucidate the complex genetic networks governing weed herbicide responses, highlighting the need for integrated multi-omics analyses.

Building upon successful multi-omics applications in annual crops, we propose an adapted framework for tea plantation weed management that addresses three critical research directions: (1) establishing comprehensive omics resources for tea-associated weeds through chromosome-level genome assemblies to enable comparative genomics and evolutionary analysis of resistance mechanisms under tea cultivation pressures; (2) developing stage-specific multi-omics profiling that accounts for unique temporal aspects of tea systems, including weed seed bank dynamics under continuous canopy cover, competition patterns during flushing cycles, and metabolic adaptation to tea allelochemicals; (3) implementing innovative applications such as single-cell omics for meristem targeting, microbiome manipulation for ecological weed suppression, and portable sequencing for resistance monitoring.

### Herbicide-tolerant tea plant: Epigenome editing strategies for improvement

Epigenome editing is emerging as a significant advancement in plant genetic improvement. Among these approaches, CRISPR/dCas9-based epigenome editing offers novel strategies for developing herbicide-resistant (HR) crops. Unlike conventional gene editing, the CRISPR/dCas9 system enables reversible regulation of gene expression without altering the genetic code, thereby facilitating precise control of herbicide resistance traits [249,250]. Current research proposes multiple dCas9-based strategies to confer herbicide resistance in crops, including conditional silencing of herbicide-sensitive genes, activation of detoxification pathways, and modulation of herbicide transport. The remarkable success of CRISPR/dCas9-based epigenome editing in developing herbicide-resistant (HR) varieties of annual crops (*Oryza sativa*, *Triticum aestivum*, *Zea mays*) provides a compelling roadmap for its application in perennial woody species like *Camellia sinensis*.

Nevertheless, CRISPR/dCas9 development in tea plant domain remains limited, and critical considerations must be addressed to achieve successful technology transfer. Unlike herbaceous model plants, tea plant presents unique biological constraints that must be addressed, including its long generation time (3–5 years to maturity), complex secondary metabolite pathways that may interact with herbicide detoxification, and perennial growth habit requiring stable, heritable epigenetic modifications. To overcome these challenges, research should prioritize (1) target identification through comprehensive characterization of tea acetolactate synthase (ALS) isoforms and their herbicide sensitivity profiles, systems biology approaches to map detoxification networks (e.g., GSTs, P450s) in tea leaves, and identification of ABC transporters governing herbicide translocation patterns; (2) tool development, including optimization of sgRNA design for tea’s heterozygous genome (2n = 30), development of tea-specific epigenetic effectors (e.g., *C. sinensis* DNMT3 orthologs), and establishment of high-efficiency protoplast and callus transformation systems; and (3) innovative delivery strategies such as nanoparticle-mediated ribonucleoprotein delivery to meristems, vascular-targeted systems exploiting tea’s xylem network, and seasonal application synchronized with cambial activity.

### Overcoming application constraints: Integrating field sensors and smart sprayers

An integrated technological pathway from field sensors to smart sprayers, incorporating precision strategies such as “digital pesticides” and “intelligent switching.” Data fusion integrated with AI—particularly machine learning and deep learning—analyzes multi-source datasets (imagery, weather, soil, historical records), performing critical tasks such as weed/pathogen classification (e.g., ML-driven hyperspectral weed detection in soybeans reducing herbicide use by > 50 %), outbreak prediction, and generation of optimized “digital pesticide” prescriptions. These prescriptions synthesize sensor inputs with agronomic models, economic thresholds, and regulatory constraints for site-specific targeting [251]. Smart sprayers execute prescriptions via intelligent spray control systems, combining real-time AI-assisted target detection (e.g., high-speed cameras), nozzle-level ON/OFF actuation, and variable rate technology (VRT) for dynamic volume adjustment. Drift reduction technologies (DRT) and automated section control (ASC) minimize off-target impacts. Integrated telematics document application parameters for compliance and analytics, as demonstrated in large-scale operations and recent AI-sprayer integrations that significantly reduced drift in rice paddies [252,253].

The demonstrated success of smart spraying systems in annual crops provides a transformative framework for tea plantation management, though significant adaptations are required to address perennial crop challenges. Key implementation strategies must include: (1) developing tea-specific spectral libraries accounting for persistent woody structure interference and dynamic flushing patterns, with UAV protocols optimized for mountainous terrain and multi-layered canopy monitoring; (2) retraining AI models using curated tea-weed image repositories that capture growth-stage specific competition patterns and differentiate tea stress symptoms; (3) modifying sprayer hardware with canopy-penetrating nozzles and terrain-compensating boom systems tailored to dense tea bushes. Successful translation will require overcoming unique barriers including economic viability for smallholders, technical training needs, and regulatory adaptation for perennial crop applications.

## Conclusion

While chemical herbicides remain the dominant control strategy due to their immediate efficacy, their ecological and agronomic costs—including soil degradation, water contamination, and biodiversity loss—demand urgent innovation. Addressing these challenges requires moving from a focus on eradication toward a model of intelligent coexistence that balances effectiveness, yield, and sustainability.

In this context, eco-friendly weed management in tea systems can be understood through three interconnected dimensions: maintaining efficacy by suppressing dominant weeds, sustaining yields by reducing resource competition, and ensuring long-term environmental resilience by minimizing chemical inputs and conserving biodiversity. Achieving this balance calls for integrated strategies that combine chemical, microbial, and technological innovations.

Building on this foundation, the future of sustainable tea plantation weed management will increasingly rely on advanced technologies. The integration of AI, functional genomics, and synthetic biology enables the high-throughput screening of herbicides to identify novel phytotoxins and optimize herbicide design. Machine learning models trained on weed genomics and herbicide interactions can predict species-specific molecular targets, minimizing off-target effects. Advanced delivery systems, such as nano-biohybrid carriers (e.g., lignin-based nanoparticles or DNA origami) and drone-swarm precision spraying, enhance spatiotemporal accuracy while reducing herbicide volumes. Additionally, CRISPR-dCas9-mediated epigenetic editing and allelopathic tea cultivars offer ecological weed management strategies by transiently boosting crop resilience or suppressing weed growth. To ensure adoption, blockchain-enabled stewardship and decentralized bioherbicide production can align technological innovation with socioeconomic needs, promoting sustainable tea agroecosystems.

## Compliance with ethics requirements

This article does not contain any studies with human or animal subjects.

## CRediT authorship contribution statement

**Lan Chen:** Writing – original draft, Investigation, Conceptualization. **Xiaolong Yang:** Data curation. **Zhongzeng Su:** Data curation. **Xiong Guan:** Writing – review & editing, Supervision, Project administration. **Zixuan Wang:** Investigation, Supervision. **Tianpei Huang:** Writing – review & editing, Supervision, Project administration.

## Declaration of competing interest

The authors declare that they have no known competing financial interests or personal relationships that could have appeared to influence the work reported in this paper.
