# Supplementary material for: Sustainable tea plantations: Harnessing chemical-microbial synergy and smart application triangulation for targeted weed control
Source: J Adv Res. 2025 Oct 30;85:189–211. doi: 10.1016/j.jare.2025.10.054 (PMC13316500; doi:10.1016/j.jare.2025.10.054)
Supplement: Supplementary Data 1 [file mmc1.docx]

**Supplementary Table S1**

Classification and ecological traits of dominant weed species in tea plantations

| Family | Species | Life form | Frequency (%) |
| --- | --- | --- | --- |
| Equisetaceae | *Equisetum ramosissimum*^★★^ | Overground branches perennial | 17.65 |
|  | *Equisetum arvense*^—^ | Annual herb | 5.88 |
| Lygodiaceae | *Lygodium japonicum*^★★^ | Perennial climbing herb | 41.18 |
| Dennstaedtiaceae | *Pteridium aquilinum* var. *latiusculum*^★★^ | Perennial herb | 23.53 |
| Poaceae | *Setaria viridis*^☆^ | Annual herb | 52.94 |
|  | *Cynodon dactylon*^—^ | Perennial herb | 29.41 |
|  | *Eleusine indica*^★★^ | Annual herb | 52.94 |
|  | *Arthraxon hspidus*^★^ | Annual herb | 29.41 |
|  | *Alopecurus aequalis*^☆^ | Annual herb | 29.41 |
|  | *Digitaria sanguinalis*^★^ | Annual herb | 47.06 |
|  | *Leptochloa chinensis*^—^ | Annual herb | 11.76 |
|  | *Panicum bisulcatum*^—^ | Annual herb | 11.76 |
|  | *Poa annua*^★★^ | Annual or winter herb | 29.41 |
| Cyperaceae | *Cyperus rotundus*^★★^ | Perennial herb | 47.06 |
| Cannabaceae | *Humulus scandens*^—^ | Perennial climbing herb | 17.65 |
| Polygonaceae | *Polygonum hydropiper*^★^ | Annual erecting shrub | 17.65 |
|  | *Polygonum senticosum*^★★^ | Annual herb | 17.65 |
|  | *Polygonum lapathifolium*^★^ | Annual herb | 29.41 |
| Phytolaccaceae | *Phytolacca acinosa*^★★^ | Perennial herb | 17.65 |
| Portulacaceae | *Portulaca oleracea*^—^ | Annual herb | 29.41 |
| Caryophyllaceae | *Stellaria media*^★★^ | Annual or biennial herb | 58.82 |
| Amaranthaceae | *Achyranthes bidentata*^—^ | Perennial herb | 41.18 |
| Brassicaceae | *Cardamine hirsuta*^☆^ | Annual herb | 29.41 |
| Rosaceae | *Rubus hirsutus*^★★^ | Perennial herb | 23.53 |
|  | *Rubus corchorifolius*^★★^ | Perennial erecting shrub | 17.65 |
| Oxalidaceae | *Oxalis corniculata*^★^ | Perennial herb | 58.82 |
| Geraniaceae | *Geranium carolinianum*^★★^ | Perennial herb | 47.06 |
| Euphorbiaceae | *Acalypha australis*^—^ | Annual herb | 41.18 |
|  | *Euphorbia helioscopia*^★★^ | Annual herb | 35.29 |
|  | *Euphorbia humifusa*^☆^ | Annual herb | 29.41 |
| Apocynaceae | *Trachelospermum jasminoides*^—^ | Perennial vine | 23.53 |
| Rubiaceae | *Galium aparine* var. *tenerum*^☆^ | Branched, trailing, or climbing herb | 35.29 |
| Convolvulaceae | *Ipomoea purpurea*^★★^ | Annual twining herb | 17.65 |
| Boraginaceae | *Trigonotis peduncularis*^—^ | Biennial herb | 41.18 |
| Solanaceae | *Solanum nigrum*^★★^ | Annual herb | 41.18 |
|  | *Solanum lyratum*^—^ | Perennial vine | 11.76 |
| Asteraceae | *Xanthium sibiricum*^—^ | Annual herb | 41.18 |
|  | *Hemistepta lyrata*^—^ | Annual herb | 29.41 |
|  | *Taraxacum mongolicum*^—^ | Perennial herb | 35.29 |
|  | *Emilia sonchifolia*^☆^ | Annual herb | 35.29 |

Note: ^★★^ represents malignant weeds, ^★^ corresponds to general weeds, ^☆^ denotes harmless weeds, and ^−^ designates unclassified weeds. Occurrence frequency (%) is defined as the ratio of a weed's documented appearances across seventy referenced studies to the total number of surveyed literature sources, multiplied by 100%. This metric provides a quantitative measure of weed prevalence within the surveyed area.
